# Terapias Modificadoras de Doença para a Cardiomiopatia por Amiloidose por Transtirretina: Uma Revisão Sistemática e Metanálise

**DOI:** 10.36660/abc.20240830

**Published:** 2025-09-10

**Authors:** Ligia Carolina Facin, Igor Pacheco Fiuza Romeiro, Kaushiki Sapahia, Beatriz Austregésilo de Athayde de Hollanda Morais, Juliana Queiroz Vasconcelos Muniz, Juliana Domenes Pereira, Cintia Gomes, Celina Borges Migliavaca, André Zimerman, Andreia Biolo

**Affiliations:** 1 Hospital Moinhos de Vento Serviço de Cardiologia Porto Alegre RS Brasil Serviço de Cardiologia, Hospital Moinhos de Vento, Porto Alegre, RS – Brasil; 2 Universidade Federal do Ceará Departamento de Medicina Fortaleza PE Brasil Departamento de Medicina, Universidade Federal do Ceará, Fortaleza, PE – Brasil; 3 University College of Medical Sciences of New Delhi Department of Medicine Delhi Índia Department of Medicine, University College of Medical Sciences of New Delhi, Delhi – Índia; 4 Centro Universitário de Maceió Departamento de Medicina Maceió AL Brasil Departamento de Medicina, Centro Universitário de Maceió (Cesmac), Maceió, AL – Brasil; 5 Schmieder Klinik Heidelberg Department of Internal Medicine Heidelberg Alemanha Department of Internal Medicine, Schmieder Klinik Heidelberg, Heidelberg – Alemanha; 6 Universidade Estadual de Campinas Departamento de Clínica Médica São Paulo SP Brasil Departamento de Clínica Médica, Universidade Estadual de Campinas, São Paulo, SP – Brasil; 7 University of Colorado Health Parkview Medical Center Department of Internal Medicine Colorado EUA Department of Internal Medicine, University of Colorado Health Parkview Medical Center, Colorado – EUA; 8 Universidade Federal do Rio Grande do Sul Programa de Pós-Graduação em Cardiologia Porto Alegre RS Brasil Programa de Pós-Graduação em Cardiologia, Universidade Federal do Rio Grande do Sul, Porto Alegre, RS – Brasil; 9 Hospital Moinhos de Vento MOVE Academic Research Organization Porto Alegre RS Brasil MOVE Academic Research Organization, Hospital Moinhos de Vento, Porto Alegre, RS - Brasil; 10 Hospital de Clínicas de Porto Alegre Serviço de Cardiologia Porto Alegre RS Brasil Serviço de Cardiologia, Hospital de Clínicas de Porto Alegre, Porto Alegre, RS – Brasil

**Keywords:** Cardiomiopatia por Amiloidose por Transtirretina, Estabilizador de TTR, Silenciador de TTR

## Abstract

**Fundamento:**

A cardiomiopatia por amiloidose por transtirretina (ATTR-CM) é a forma mais comum de cardiomiopatia restritiva. Novas terapias farmacológicas buscam modificar a progressão natural da doença e retardar seu avanço. No entanto, ainda são escassos os dados que comparam diretamente a eficácia das diferentes classes de fármacos em relação ao placebo.

**Objetivos:**

Esta revisão sistemática e metanálise avaliou a eficácia dos estabilizadores e silenciadores de transtirretina (TTR), em comparação ao placebo, sobre a mortalidade e hospitalizações por todas as causas, desfechos funcionais e níveis do biomarcador NT-proBNP em pacientes com ATTR-CM.

**Métodos:**

Foram realizadas buscas nas bases de dados PubMed, Embase e Cochrane por ensaios clínicos randomizados (ECRs) publicados até abril de 2025, que avaliaram patisiran, tafamidis, inotersen, revusiran, acoramidis ou vutrisiran versus placebo em pacientes com ATTR-CM. As análises foram estratificadas por classe de fármaco, considerando significância estatística para p<0,05.

**Resultados:**

Foram incluídos sete ECRs, totalizando 2.526 participantes; 42,5% receberam estabilizadores de TTR e 57,5% receberam silenciadores de TTR. Em comparação ao placebo, os estabilizadores de TTR reduziram significativamente a mortalidade (RR: 0,71; IC 95% 0,59-0,87; p=0,0006) e e hospitalizações (RR: 0,81; IC 95% 0,73-0,89; p<0,0001), ambas por todas as causas. Já os silenciadores de TTR não reduziram significativamente nem a mortalidade (RR: 0,79; IC 95% 0,37-1,68; p=0,54) nem as hospitalizações (RR: 1,11; IC 95% 0,83-1,48; p=0,48). As duas abordagens terapêuticas melhoraram a distância percorrida no teste de caminhada de 6 minutos, a qualidade de vida e reduziram os níveis séricos NT-proBNP.

**Conclusão:**

Os estabilizadores de TTR reduziram significativamente a mortalidade e as hospitalizações por todas as causas em pacientes com ATTR-CM, em comparação ao placebo. Esses benefícios não foram observados com os silenciadores de TTR, possivelmente em função do tempo de seguimento mais curto nos estudos incluídos. Ambas as terapias, contudo, promoveram melhorias no estado funcional e na redução dos níveis séricos do biomarcador NT-proBNP.

**Figure:**
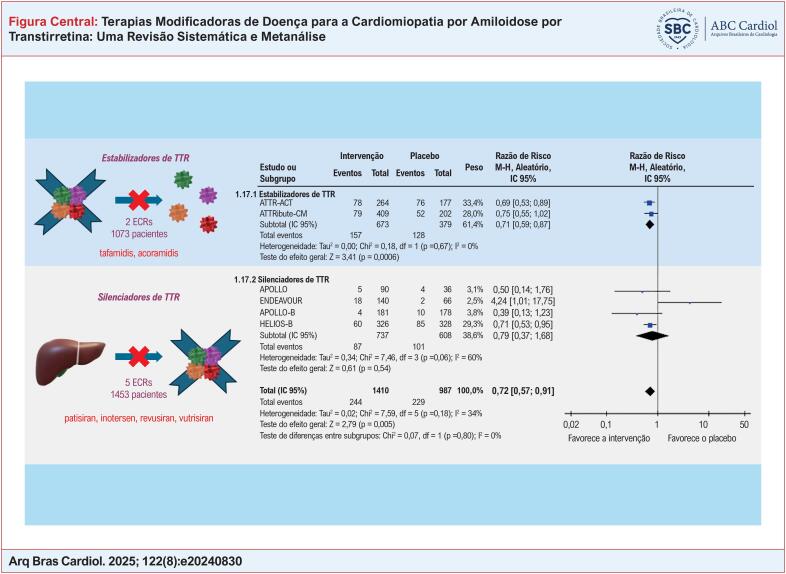
Os estabilizadores de TTR previnem a dissociação da proteína transtirretina, enquanto os silenciadores de TTR inibem sua produção, atuando principalmente no citoplasma das células hepáticas. Os dados indicam que os estabilizadores de TTR reduzem significativamente a mortalidade por todas as causas em comparação ao placebo em pacientes com ATTR-CM. ATTR-CM: cardiomiopatia por amiloidose por transtirretina; Chi^2^: qui-quadrado; IC: intervalo de confiança; df: graus de liberdade; I^2^: estatística I^2^ de Higgins; M-H: método de Mantel-Haenszel; p: valor de p; Tau: tau de Kendall; TTR: transtirretina.

## Destaques

- As terapias modificadoras de doença para ATTR-CM estão disponíveis e podem retardar a progressão da doença e melhorar o prognóstico;
- Nesta metanálise de ECRs, os estabilizadores de TTR reduziram significativamente a mortalidade e hospitalizações por todas as causas, confirmando sua eficácia na ATTR-CM;
- Os silenciadores de TTR não demonstraram efeitos significativos sobre a mortalidade e as hospitalizações em comparação ao placebo; esses resultados podem ter sido influenciados pelo tempo de seguimento mais curto nos estudos disponíveis;
- Ensaios clínicos em andamento com silenciadores de TTR e novas classes de fármacos deverão fornecer evidências adicionais e complementar os achados desta metanálise.

## Introdução

A amiloidose cardíaca (AC) apresenta uma incidência estimada entre 18 e 55 casos por 100.000 pessoas-ano, com aumento progressivo tanto na prevalência quanto na mortalidade associada nas últimas décadas.^1^ No Reino Unido, o número de pacientes diagnosticados com amiloidose aumentou 6,7 vezes desde a década de 1990, refletindo, provavelmente, uma maior conscientização sobre a doença e os avanços nas técnicas diagnósticas. Nos Estados Unidos, as taxas de mortalidade também aumentaram de forma significativa, passando de 1,77 por 1.000.000 em 1979 para 3,96 por 1.000.000 em 2015.^2^ A AC é uma condição progressiva que pode ser fatal caso não seja diagnosticada e tratada precocemente.^3 - 5^

As terapias convencionais atuam apenas sobre as manifestações clínicas, como arritmias ou insuficiência cardíaca, mas não interrompem a progressão da doença.^6 , 7^ O desenvolvimento de terapias modificadoras de doença transformou o manejo da cardiomiopatia por amiloidose por transtirretina (TTR) (ATTR-CM). A TTR é uma proteína plasmática solúvel de curta meia-vida, sintetizada principalmente pelo fígado, com estrutura tetramérica composta por quatro subunidades idênticas, responsáveis pelo transporte de tiroxina e vitamina A. As terapias mais estudadas e clinicamente disponíveis direcionadas à cardiomiopatia amiloide são os estabilizadores e os silenciadores de TTR ( Figura Central ).^8 - 10^ Os estabilizadores de TTR, incluindo tafamidis e acoramidis, ligam-se à interface dímero-dímero do tetrâmero de TTR, impedindo sua dissociação em monômeros e, consequentemente, a formação de fibrilas amiloides que se depositam no miocárdio.^11^ Por outro lado, os silenciadores de TTR atuam predominantemente no citoplasma dos hepatócitos, inibindo a síntese da proteína TTR e reduzindo de forma significativa as concentrações circulantes de TTR.^12^ Esta classe inclui oligonucleotídeos antisense (p. ex., inotersen) e RNAs de interferência curta (p.ex., revusiran, patisiran e vutrisiran).

Até o momento, nenhum estudo sintetizou de forma abrangente a eficácia das terapias direcionadas à ATTR-CM, especialmente quando estratificadas por mecanismo de ação.^13 , 14^ Esta revisão teve como objetivo avaliar a eficácia e a segurança das terapias modificadoras de doença para ATTR-CM, incluindo tafamidis, revusiran, patisiran, inotersen, acoramidis e vutrisiran, em comparação ao placebo. A análise teve como foco desfechos centrados no paciente, incluindo mortalidade e hospitalizações por todas as causas, capacidade funcional, qualidade de vida, além dos níveis séricos de NT-proBNP.

## Métodos

### Protocolo

Esta revisão foi prospectivamente registrada no *International Prospective Register of Systematic Reviews* (PROSPERO) sob o número de protocolo CRD42024517136.

### Desenho do estudo

Esta revisão foi conduzida de acordo com as diretrizes do *Cochrane Handbook for Systematic Reviews of Interventions* e reportada conforme as recomendações do *Preferred Reporting Items for Systematic Reviews and Meta-Analyses* (PRISMA).^15 , 16^

### Estratégia de busca, critérios de elegibilidade e extração de dados

Foi realizada uma busca sistemática nas bases de dados PubMed, Embase e Cochrane em 13 de abril de 2025. A estratégia de busca completa está apresentada na Tabela S1 . Após a remoção dos duplicados, a seleção dos estudos foi realizada em duas fases. Na primeira, títulos e resumos foram triados utilizando o Zotero. Em seguida, os textos completos dos estudos potencialmente elegíveis foram avaliados para inclusão. Dois revisores independentes (L.F. e C.G.) realizaram o processo de seleção, e eventuais divergências foram resolvidas por um terceiro revisor (A.B.).

Os critérios de inclusão foram: (1) ensaios clínicos randomizados (ECRs) revisados por pares; (2) avaliação de terapias modificadoras de doença para ATTR-CM em pacientes com envolvimento cardíaco confirmado; e (3) relato de pelo menos um desfecho de interesse. Os estudos foram excluídos se: (1) incluíssem populações duplicadas; (2) não tivessem grupo controle; ou (3) não apresentassem desfechos relevantes. Não foram aplicadas restrições quanto à data de publicação, idioma ou duração do seguimento.

Foram extraídos dados sobre o desenho dos estudos (duração do seguimento, intervenção e número de pacientes randomizados) e características basais dos pacientes (sexo, idade, classe funcional da New York Heart Association [NYHA], pró-peptídeo natriurético cerebral N-terminal [NT-proBNP] e fração de ejeção do ventrículo esquerdo [VE] [FEVE]).

Dois revisores independentes (L.F. e I.P.) realizaram a extração dos dados utilizando uma planilha do Excel previamente elaborada para esta revisão.

### Desfechos de interesse

Os desfechos de interesse incluíram mortalidade por todas as causas, mortalidade cardiovascular, hospitalizações por todas as causas, hospitalizações cardiovasculares, hospitalizações por insuficiência cardíaca, capacidade funcional avaliada pelo teste de caminhada de 6 minutos (TC6M), qualidade de vida avaliada pelo *Kansas City Cardiomyopathy Questionnaire* – *Overall Score* (KCCQ-OS), níveis séricos de NT-proBNP e de transtirretina (TTR), *strain* longitudinal global (GLS) do VE, massa do VE, espessura da parede do VE e FEVE. A disponibilidade de cada desfecho nos estudos incluídos está detalhada na Tabela S2 .

### Análise dos dados e estatística

Foram realizadas metanálises com modelo de efeitos aleatórios, utilizando o método de Mantel-Haenszel ou o método de Variância Inversa Genérica para desfechos binários, e o método de variância inversa para desfechos contínuos, com a estimativa de Tau realizada pelo método de DerSimonian e Laird.^16^ Quando necessário, os desvios padrão das variações foram imputados utilizando um coeficiente de correlação de 0,5.^16^ A heterogeneidade foi avaliada por meio da estatística I^2^ de Higgins e explorada adicionalmente por meio de análises de subgrupos.

Todas as análises estatísticas foram realizadas no software RevMan, versão 5.4 (Nordic Cochrane Centre, The Cochrane Collaboration, Copenhague, Dinamarca).^17^ Foi considerado estatisticamente significativo um valor de p bilateral <0,05.

Adicionalmente, para considerar variações na duração do seguimento, as mudanças anualizadas no TC6M e no KCCQ-OS foram calculadas dividindo-se a variação absoluta pelo tempo de seguimento relatado em cada estudo.

### Avaliação da qualidade

O risco de viés dos estudos incluídos foi avaliado utilizando a ferramenta *Risk of Bias 2* (RoB 2) para ECRs.^18^ A certeza da evidência para cada desfecho foi avaliada de acordo com o *Grading of Recommendations, Assessment, Development and Evaluation* (GRADE).^19^

## Resultados

### Seleção dos estudos e características basais

**Figura 1:**
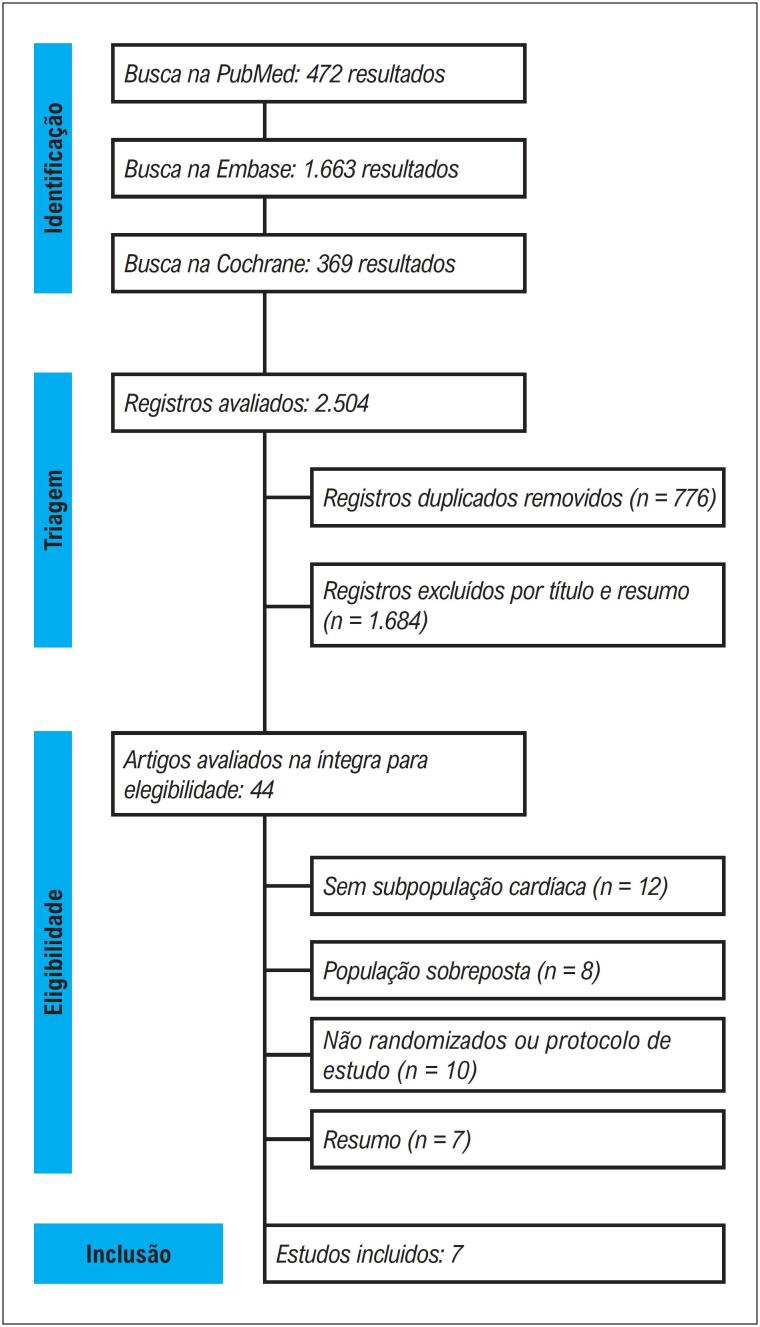
Diagrama de fluxo PRISMA para triagem e seleção de estudos.

O processo de seleção dos estudos está ilustrado na Figura 1 . Foram identificadas 2.504 referências por meio das buscas nas bases de dados. Após a remoção de duplicatas e a triagem de títulos e resumos, 44 artigos foram selecionados para avaliação do texto completo. Ao final, sete ECRs foram incluídos, relatados em 11 publicações, incluindo quatro análises post hoc. Esses ECRs incluíram um total de 2.526 pacientes, sendo 1.073 (42,5%) nos estudos que avaliaram estabilizadores de TTR e 1.453 (57,5%) nos estudos que avaliaram silenciadores de TTR.

As características basais dos pacientes estão resumidas na Tabela 1 . Os sete ECRs incluídos avaliaram patisiran, inotersen, tafamidis, revusiran, acoramidis e vutrisiran. A maioria dos participantes era do sexo masculino (89%). Em todos os estudos, a FEVE média ou mediana foi consistentemente ≥50%, e os níveis medianos de NT-proBNP estavam uniformemente elevados. Além disso, a maior parte dos pacientes (86%) foi classificada como classe funcional I ou II da NYHA. A duração do seguimento variou de aproximadamente 6 a 36 meses.

**Tabela 1:** Características basais dos estudos incluídos

ID do estudo	Intervenção	População principal	Classe de TTR	Pacientes (Int/Pla)	Idade a (Int/Pla)	Mulheres (%) (Int/Pla)	NYHA < III (%)(Int/Plac)	NT-proBNP b (pg/ml) (Int/Pla)	FEVE a (%) (Int/Pla)	Período de acompanhamento
Adams 2018 (APOLLO)^12^	Patisiran	ATTR-PN	Silenciador	90/36	60/62	24/18	100/100	756,4/845,7	60/62	18 meses
Benson 2018 (NEURO-TTR)^28^	Inotersen	ATTR-PN	Silenciador	75/33	NA d	NA d	100/100	NA d	65/64	15 meses
Maurer 2018 (ATTR-ACT)^22^	Tafamidis	ATTR-CM	Estabilizador	264/177	74/74	9/11	70/65	2.995,9/3.161,0	48/49	30 meses
Judge 2020 (ENDEAVOUR)^20^	Revusiran	ATTR-CM	Silenciador	140/66	69/68	25/20	69/70	2.371,0/2.719,0	53/51	6.7 meses c
Maurer 2023 (APOLLO-B)^29^	Patisiran	ATTR-CM	Silenciador	181/178	76/76	11/10	92/93	2.008,0/1.813,0	58/60	12 meses
Gilmore 2024 (ATTRibute-CM)^21^	Acoramidis	ATTR-CM	Estabilizador	421/211	77/77	9/12	89/90	2.326,0/2.306,0	NA e	30 meses
Fontana 2024 (HELIOS-B)^23^	Vutrisiran	ATTR-CM	Silenciador	326/328	77/76	8/7	92/90	2.021,0/1.801,0	NA e	36 meses

a Média ou mediana;

b Mediana;

c Mediana;

d Dados não disponíveis para pacientes com cardiomiopatia;

e Dados não disponíveis.

ATTR-PN: amiloidose por transtirretina com polineuropatia; ATTR-CM: cardiomiopatia amiloide por transtirretina; Int: grupo de intervenção; Pla: grupo placebo; NYHA: New York Heart Association; NT-proBNP: pró-peptídeo natriurético cerebral N-terminal; LVEF: fração de ejeção do ventrículo esquerdo; NA: não disponível. Todos os estudos adotaram nível de significância estatística de 5% (p < 0,05).

### Avaliação da qualidade

De modo geral, a maioria dos desfechos foi classificada como apresentando baixo risco de viés. No entanto, diversos desfechos do estudo ENDEAVOUR^20^ foram avaliados como de alto risco de viés ou com algumas preocupações, principalmente devido a questões relacionadas à mensuração dos desfechos e dados ausentes. A avaliação detalhada do risco de viés está apresentada na Figura S1 .

A certeza da evidência para cada desfecho foi classificada como muito baixa, baixa, moderada ou alta, conforme apresentado na Tabela S3 . As limitações mais frequentemente identificadas foram inconsistência e imprecisão.

### Mortalidade e hospitalizações

**Figura 2:**
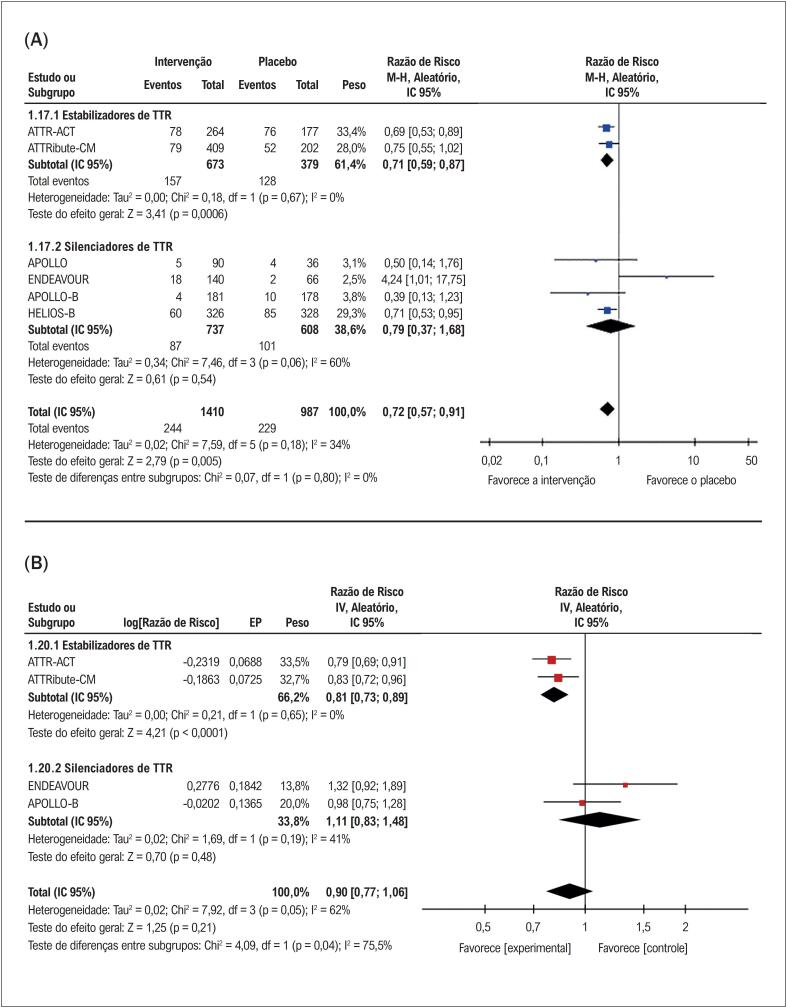
(A) Mortalidade por todas as causas com terapias modificadoras da doença versus placebo em pacientes com ATTR-CM. (B) Hospitalização por todas as causas com terapias modificadoras da doença versus placebo em pacientes com ATTR-CM. Chi^2^: qui-quadrado; EP: erro padrão; IC: intervalo de confiança; df: graus de liberdade; I-quadrado: estatística I-quadrado de Higgins; IV: variância inversa; log: logaritmo; M-H: Mantel-Haenszel; p: valor de p; Tau: tau de Kendall; TTR: transtirretina.

Conforme ilustrado na Figura 2A , ao agrupar todos os estudos, observou-se uma redução significativa na mortalidade por todas as causas (RR: 0,72; IC 95%, 0,57-0,91; p=0,005). Na análise de subgrupos, os estabilizadores de TTR reduziram significativamente a mortalidade por todas as causas em comparação ao placebo (RR: 0,71; IC 95% 0,59-0,87; p=0,0006), enquanto nenhuma diferença significativa foi observada no subgrupo de silenciadores de TTR (RR: 0,79; IC 95% 0,37-1,68; p=0,54). No que diz respeito à mortalidade cardiovascular, os dados disponíveis limitaram-se aos estudos que avaliaram silenciadores de TTR, os quais não demonstraram redução significativa no risco em comparação ao placebo (RR: 1,87; IC 95% 0,64-5,44; p=0,25). Apesar desses achados em relação à mortalidade por todas as causas, não foram observadas diferenças significativas entre as classes de fármacos e o placebo ao se analisar o valor de p para interação.

Como demonstrado na Figura 2B , ao combinar todas as terapias, não foi observado efeito significativo sobre as hospitalizações por todas as causas (RR: 0,90; IC 95% 0,77-1,06; p=0,21). No entanto, na análise de subgrupos, os estabilizadores de TTR reduziram significativamente as taxas de hospitalização em comparação ao placebo (RR: 0,81; IC 95% 0,73-0,89; p<0,0001), enquanto os silenciadores de TTR não apresentaram efeito significativo (RR: 1,11; IC 95% 0,83-1,48; p=0,48). Para hospitalizações de causa cardíaca, não foi possível realizar metanálise por classes terapêuticas, sendo a análise restrita ao conjunto de todas as intervenções, sem significância estatística (RR: 0,90; IC 95%: 0,75–1,09; p = 0,28). Quanto às hospitalizações por insuficiência cardíaca, a metanálise foi viável para a análise geral (RR: 1,03; IC 95%: 0,69–1,54; p = 0,88) e para o subgrupo de silenciadores de TTR (RR: 1,40; IC 95%: 0,84–2,34; p = 0,19) somente, ambas tambem sem diferenças significativas.

### Capacidade funcional e qualidade de vida

**Figura 3:**
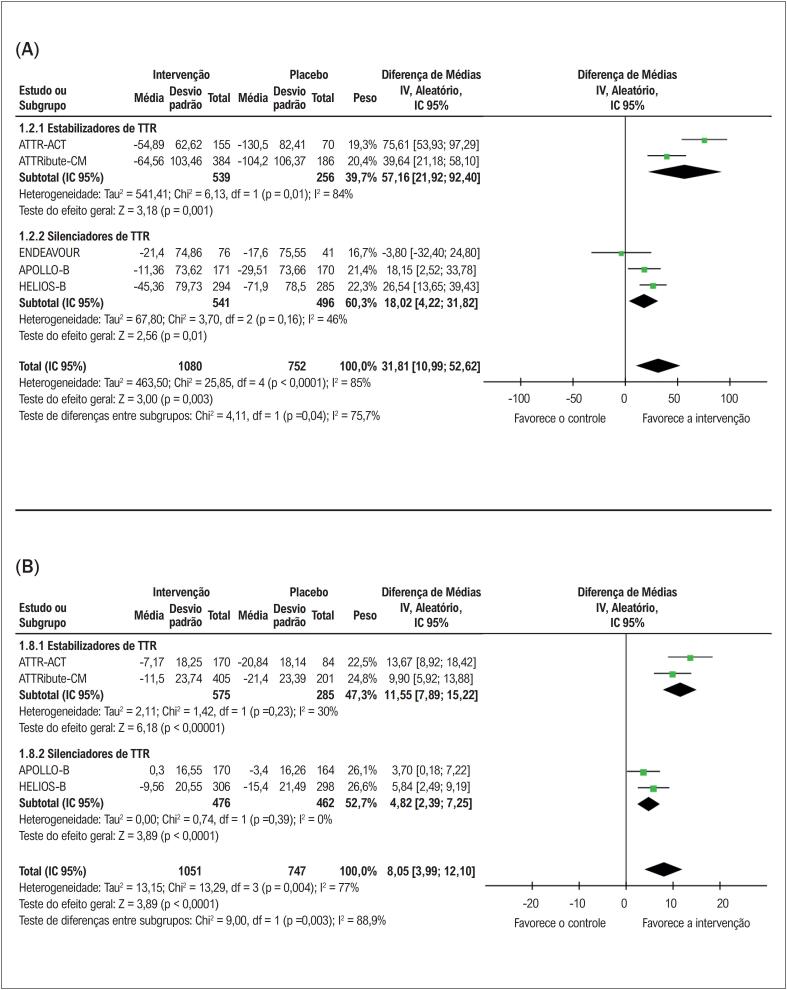
(A) Teste de caminhada de 6 minutos (TC6M) com terapias modificadoras da doença versus placebo em pacientes com ATTR-CM. (B) Kansas City Cardiomyopathy Questionnaire Overall Score (KCCQ-OS) com terapias modificadoras da doença versus placebo em pacientes com ATTR-CM. DTP6: distância percorrida em 6 minutos; Chi^2^: qui-quadrado; IC: intervalo de confiança; df: graus de liberdade; I^2^: estatística I^2^ de Higgins; IV: variância inversa; p: valor de p; Tau: tau de Kendall; TTR: transtirretina; KCCQ-OS: Kansas City Cardiomyopathy Questionnaire Overall Score.

Como demonstrado na Figura 3A , o tratamento com terapias modificadoras de doença resultou em melhora significativa na distância percorrida no TC6M em comparação ao placebo (diferença média [DM] de 31,81 metros; IC 95%, 10,99-52,62; p=0,003). De maneira semelhante, a Figura 3B mostra uma melhora significativa nos escores do KCCQ-OS (DM de 8,05 pontos; IC 95%, 3,99-12,10; p<0,0001).

Na análise de subgrupos, a melhora no TC6M foi significativa em ambos os grupos: estabilizadores de TTR (DM: 57,16 metros; IC 95%, 21,92-92,40; p=0,001) e silenciadores de TTR (DM: 18,02 metros; IC 95%, 4,22-31,82; p=0,01). Quanto ao KCCQ-OS, ambos os subgrupos também apresentaram melhorias significativas: estabilizadores de TTR (DM: 11,55 pontos; IC 95%, 7,89-15,22; p<0,00001) e silenciadores de TTR (DM de 4,82 pontos; IC 95%, 2,39-7,25; p<0,0001).

### Desfechos laboratoriais

**Figura 4:**
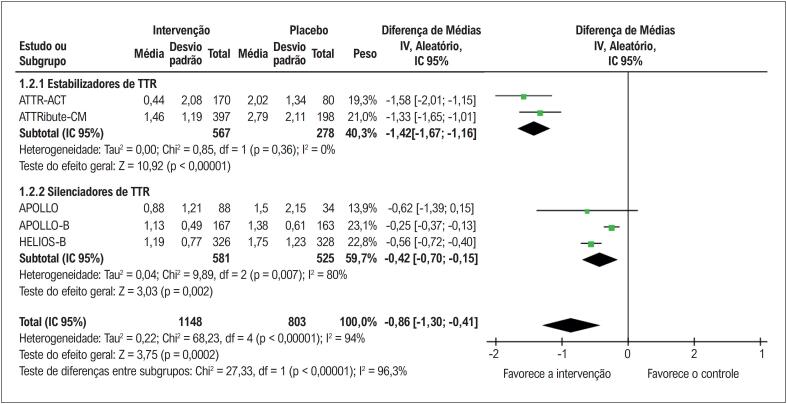
Níveis séricos de NT-proBNP com terapias modificadoras da doença versus placebo em pacientes com ATTR-CM. Chi^2^: qui-quadrado; IC: intervalo de confiança; df: graus de liberdade; I^2^: estatística I^2^ de Higgins; IV: variância inversa; NT-proBNP: pró-peptídeo natriurético cerebral N-terminal; p: valor-p; Tau: tau de Kendall; TTR: transtirretina.

Conforme apresentado na Figura 4 , o tratamento com terapias modificadoras de doença resultou em uma redução significativa nos níveis de NT-proBNP em comparação ao placebo (DM na variação geométrica em relação ao basal: −0,86 pg/ml; IC 95% −1,30 a −0,41; p=0,0002). De maneira semelhante, a Figura S2 demonstra uma redução significativa nos níveis séricos de proteína TTR em comparação ao placebo (DM: −70,25%; IC 95% −97,04 a −43,46; p<0,00001).

Na análise de subgrupos por classe terapêutica, tanto os estabilizadores de TTR (DM na variação geométrica em relação ao basal: −1,42 pg/ml; IC 95% −1,67 a −1,16; p<0,00001) quanto os silenciadores de TTR (DM na variação geométrica em relação ao basal: −0,42 pg/ml; IC 95% −0,70 a −0,15; p=0,002) reduziram significativamente os níveis de NT-proBNP. Em relação aos níveis séricos de TTR só foi possivel realizar metanalise do subgrupo dos silenciadores de TTR, que apresentaram redução significativa (DM: −81,62%; IC 95% −86,44 a −76,80; p<0,00001).

### Desfechos ecocardiográficos

As Figuras S3A a S3D apresentam os resultados da análise dos desfechos ecocardiográficos. Embora todos os desfechos tenham sido avaliados na análise geral, apenas os estudos com silenciadores de TTR disponibilizaram dados suficientes para a condução de metanálise por subgrupo.

O uso de terapias modificadoras da doença foi associado a uma melhora significativa no GLS do VE ( Figura S3A ), em comparação ao placebo (DM: - 0,83%; IC 95%: - 1,27 a - 0,40; p = 0,0002). A análise por subgrupo demonstrou significância estatística entre os silenciadores de TTR (DM: - 0,85%; IC 95%: - 1,41 a - 0,30; p = 0,003).

Em relação à massa do VE ( Figura S3B ), observou-se uma redução significativa em comparação ao placebo (DM: - 9,74 g; IC 95%: - 17,07 a - 2,40; p = 0,009). Este desfecho foi reportado exclusivamente em estudos com silenciadores de TTR.

Não foram observadas diferenças estatisticamente significativas na espessura da parede ventricular esquerda ( Figura S3C ), tanto na análise geral (DM: - 0,30 mm; IC 95%: - 0,66 a 0,06; p = 0,10), quanto na análise por subgrupo dos silenciadores de TTR (DM: - 0,28 mm; IC 95%: - 0,67 a 0,11; p = 0,16).

De modo semelhante, a FEVE ( Figura S3D ) não apresentou diferença significativa entre os grupos intervenção e controle (DM: - 0,16%; IC 95%: - 1,97 a 1,64; p = 0,86), e também não foi identificado efeito significativo na análise por subgrupo dos silenciadores de TTR (DM: −0,99%; IC 95%: - 3,05 a 1,06; p = 0,34).

### Análise de sensibilidade

Foi realizada uma análise de sensibilidade excluindo o estudo ENDEAVOUR,^20^ devido à sua interrupção precoce. Esta análise revelou uma redução significativa na mortalidade por todas as causas (RR: 0,70; IC 95% 0,60-0,82; p<0,0001) ( Figura S4A ). Na análise de subgrupos, tanto os estabilizadores de TTR (RR: 0,71; IC 95% 0,59-0,87; p=0,0006) quanto os silenciadores de TTR (RR: 0,67; IC 95% 0,51-0,89; p=0,005) demonstraram uma redução significativa na mortalidade em comparação ao placebo.

Apesar desse achado,a análise de sensibilidade não demonstrou diferença significativa entre as classes de fármacos e o placebo ao avaliar o valor de p para interação.

No que se refere à espessura de parede de VE, a exclusão do estudo ENDEAVOUR^20^ resultou em uma redução significativa tanto na análise combinada das duas classes terapêuticas (DM –0,44 mm; IC95% –0,81 a –0,06; p=0,02) quanto no subgrupo de silenciadores de TTR (DM –0,44 mm; IC95% –0,84 a –0,03; p=0,03).

Adicionalmente, foi realizada uma análise de sensibilidade, excluindo os estudos cujo objetivo principal era o tratamento de neuropatias amiloides. O estudo APOLLO^12^ foi removido dos desfechos considerados na análise principal - mortalidade por todas as causas ( Figura S5A ) e NT-proBNP sérico ( Figura S5B ) - a fim de confirmar que sua exclusão não alteraria a significância estatística. Para mortalidade por todas as causas, a análise global permaneceu significativa (RR 0,73; IC95% 0,57–0,94; p=0,01), enquanto nenhum efeito significativo foi observado no subgrupo de silenciadores de TTR (RR 0,92; IC95% 0,33–2,57; p=0,88). Em relação ao NT-proBNP, a significância foi mantida tanto na análise global (DM na variação geométrica em relação ao basal: –0,89 pg/mL; IC95% –1,39 a –0,40; p=0,0004) quanto no subgrupo de silenciadores de TTR (DM na variação geométrica em relação ao basal: –0,40 pg/mL; IC95% –0,70 a –0,10; p=0,010). O estudo NEURO-TTR^28^ não foi incluído nessa avaliação por disponibilizar apenas dados ecocardiográficos.

## Discussão

Esta revisão incluiu sete ECRs que compararam diversas terapias modificadoras de doença com placebo em pacientes com ATTR-CM. Os principais achados foram os seguintes: (1) os estabilizadores de TTR reduziram significativamente a mortalidade e hospitalizações por todas as causas em comparação ao placebo; (2) os silenciadores de TTR não demonstraram redução significativa nesses desfechos; e (3) ambas as classes terapêuticas foram eficazes em melhorar a capacidade funcional (TC6M) e a qualidade de vida (KCCQ-OS), bem como em reduzir os níveis séricos de NT-proBNP em comparação ao placebo.

Observamos que os estabilizadores de TTR reduziram significativamente a mortalidade em comparação ao placebo. Embora o estudo ATTRibute^21^ não tenha demonstrado uma redução estatisticamente significativa na mortalidade, os autores sugeriram que isso pode ser atribuído ao aumento da conscientização sobre a doença, resultando em diagnóstico e tratamento mais precoces, e, consequentemente, em um menor risco de mortalidade por todas as causas na população do estudo. Ao combinar os resultados dos estudos ATTR-ACT^22^ e ATTRibute^21^ em nossa revisão, o aumento do poder estatístico revelou uma redução consistente no risco de mortalidade em ambos os estudos. Além disso, os efeitos benéficos dos estabilizadores de TTR sobre a mortalidade também foram demonstrados em cenários do mundo real, reforçando a validade externa desses achados.^11^

A ausência de um efeito significativo dos silenciadores de TTR sobre a mortalidade pode ser atribuída, em parte, aos períodos de seguimento mais curtos na maioria dos estudos incluídos, resultando em menor número de eventos observados em comparação aos estudos que avaliaram estabilizadores de TTR. Nos estudos com estabilizadores de TTR, as diferenças na mortalidade entre os grupos de intervenção e placebo geralmente surgiram entre 15 e 18 meses, com ambos os ECRs apresentando duração de seguimento de 30 meses. Em contraste, o seguimento médio nos estudos com silenciadores de TTR foi de aproximadamente 17 meses. Destaca-se que o estudo HELIOS-B,^23^ que teve um seguimento mais longo, de 36 meses, demonstrou uma redução significativa na mortalidade. Além disso, ao realizar uma análise de sensibilidade excluindo o estudo ENDEAVOUR,^20^ que foi interrompido precocemente, os silenciadores de TTR também apresentaram uma redução significativa na mortalidade por todas as causas em comparação ao placebo.

As hospitalizações representam uma carga crítica no manejo da insuficiência cardíaca. Nesta análise, agrupamos dados sobre hospitalizações por todas as causas, cardiovasculares e relacionadas à insuficiência cardíaca. Nossos achados demonstraram uma redução significativa nas hospitalizações por todas as causas entre os pacientes tratados com estabilizadores de TTR em comparação ao placebo. No entanto, variações na classificação dos eventos de hospitalização entre os ECRs incluídos limitaram a possibilidade de padronizar esses dados para uma conclusão mais definitiva.

Para considerar as diferenças nas durações de seguimento — mais longas nos estudos que avaliaram estabilizadores de TTR —, foi calculada a variação anualizada, ajustada pelo placebo, no TC6M ( Tabela S4 ). Ao considerar esses resultados anualizados, ambas as classes terapêuticas demonstraram melhorias comparáveis. No entanto, as melhorias ajustadas no TC6M permaneceram abaixo do limiar de 35 metros por ano, valor sugerido como tendo significado prognóstico na AC.^24^ De maneira semelhante, para o KCCQ-OS, as variações anualizadas, ajustadas pelo placebo, foram comparáveis entre as classes terapêuticas. Ainda assim, os estabilizadores de TTR alcançaram uma melhoria superior no estudo ATTR-ACT^22^, ultrapassando o limiar de 5 pontos, associado a um impacto clínico e prognóstico significativo.^25^

Em relação aos biomarcadores,^26^ nossa análise demonstrou que as terapias modificadoras de doença estiveram associadas a reduções significativas nos níveis séricos de NT-proBNP e de proteína TTR, ambos utilizados rotineiramente para avaliar a gravidade da doença. Os estabilizadores de TTR resultaram em uma redução mais acentuada do NT-proBNP, enquanto os silenciadores de TTR promoveram uma diminuição mais pronunciada nos níveis séricos de TTR, em consonância com seu mecanismo de ação de inibição transcricional da síntese hepática de TTR. Embora os estabilizadores de TTR sejam geralmente esperados para preservar ou aumentar os níveis circulantes de TTR ao prevenir a dissociação do tetrâmero, os únicos dados disponíveis em nossa análise vieram do estudo ATTRibute-CM,^21^ que relatou uma redução. Portanto, esse achado deve ser interpretado com cautela.

A aplicabilidade dos parâmetros ecocardiográficos na avaliação do prognóstico da AC é limitada, uma vez que a maioria dos dados provém de estudos de pequeno porte. No entanto, especialmente em pacientes assintomáticos, a ecocardiografia tem se mostrado útil na detecção precoce da progressão da doença.^27^ Nossa revisão demonstrou melhorias no GLS do VE e na massa do VE, sendo a redução do GLS impulsionada principalmente pelos silenciadores de TTR. De forma semelhante, a redução da massa do VE foi observada exclusivamente nos estudos que avaliaram silenciadores de TTR. Não foram encontrados efeitos significativos sobre a espessura da parede do VE ou sobre a FEVE na análise geral. Entretanto, na análise de sensibilidade, excluindo o estudo ENDEAVOUR,^20^ observou-se significância estatística para a redução da espessura da parede do ventrículo esquerdo, tanto no efeito combinado das duas classes terapêuticas quanto na análise dos silenciadores de TTR versus placebo. É importante destacar que o tamanho da amostra para desfechos ecocardiográficos foi limitado na maioria das análises.

Metanálises anteriores^13 , 14^ focaram-se principalmente na eficácia do tafamidis, incorporando dados de ECRs e estudos observacionais. Em contraste, nossa análise incluiu sete ECRs, dos quais cinco foram especificamente desenhados para ATTR-CM. De maneira semelhante, uma metanálise conduzida por Wang et al.^13^ avaliou o efeito do tafamidis na ATTR-CM e relatou uma associação significativa entre a terapia com estabilizadores de TTR e a redução da mortalidade, em concordância com os achados do presente estudo.

Esta revisão apresenta várias limitações. Primeiramente, embora os estudos APOLLO^12^ e NEURO-TTR^28^ tenham sido desenhados principalmente para avaliar desfechos neurológicos, apenas os dados relacionados à cardiomiopatia foram incluídos em nossa análise. Uma análise de sensibilidade, excluindo o estudo APOLLO, confirmou que sua exclusão não alterou os resultados. Em segundo lugar, as durações de seguimento mais curtas nos estudos com silenciadores de TTR podem ter limitado o número de eventos clínicos observados; para mitigar essa limitação, avaliamos a a variação anualizada, ajustada pelo placebo ( Tabela S4 ). Em terceiro lugar, a interrupção precoce do estudo ENDEAVOUR^20^ pode ter comprometido a confiabilidade de seus dados; por isso, apresentamos uma análise de sensibilidade excluindo esse estudo nas análises suplementares. Quarto, não realizamos comparações diretas entre estabilizadores e silenciadores de TTR, uma vez que todas as comparações foram feitas em relação ao placebo. Por fim, os dados ecocardiográficos permanecem limitados nos estudos disponíveis; no entanto, estudos em andamento deverão fornecer evidências mais robustas.

Este estudo também apresenta diversas fortalezas, incluindo a metodologia rigorosa, a estrita adesão às diretrizes Cochrane e PRISMA, além da inclusão abrangente de todos os ECRs disponíveis que avaliaram a eficácia das terapias atualmente aprovadas para ATTR-CM.

## Conclusão

Nesta revisão sistemática e metanálise das terapias modificadoras de doença para AC, observou-se que os estabilizadores de TTR reduziram de forma significativa a mortalidade e as hospitalizações por todas as causas em pacientes com ATTR-CM em comparação ao placebo. Por outro lado, os silenciadores de TTR não demonstraram impacto significativo nesses desfechos quando comparados ao placebo. Estudos adicionais são necessários para estabelecer a eficácia em longo prazo desta classe terapêutica.

Disponibilidade de Dados

Os conteúdos subjacentes ao texto da pesquisa estão contidos no manuscrito.

## *Material suplementar

Para informação adicional, por favor, clique aqui



## References

[1] Argon A, Nart D, Yilmazbarbet F (2024). Cardiac Amyloidosis: Clinical Features, Pathogenesis, Diagnosis, and Treatment. Turk Patoloji Derg.

[2] Alexander KM, Orav J, Singh A, Jacob SA, Menon A, Padera RF (2018). Geographic Disparities in Reported US Amyloidosis Mortality From 1979 to 2015: Potential Underdetection of Cardiac Amyloidosis. JAMA Cardiol.

[3] Ruberg FL, Maurer MS (2024). Cardiac Amyloidosis Due to Transthyretin Protein: A Review. JAMA.

[4] Maurer MS, Elliott P, Comenzo R, Semigran M, Rapezzi C (2017). Addressing Common Questions Encountered in the Diagnosis and Management of Cardiac Amyloidosis. Circulation.

[5] Ioannou A, Patel RK, Razvi Y, Porcari A, Sinagra G, Venneri L (2022). Impact of Earlier Diagnosis in Cardiac ATTR Amyloidosis Over the Course of 20 Years. Circulation.

[6] Giancaterino S, Urey MA, Darden D, Hsu JC (2020). Management of Arrhythmias in Cardiac Amyloidosis. JACC Clin Electrophysiol.

[7] Brito D, Albrecht FC, Arenaza DP, Bart N, Better N, Carvajal-Juarez I (2023). World Heart Federation Consensus on Transthyretin Amyloidosis Cardiomyopathy (ATTR-CM). Glob Heart.

[8] Kittleson MM, Ruberg FL, Ambardekar AV, Brannagan TH, Cheng RK, Clarke JO (2023). 2023 ACC Expert Consensus Decision Pathway on Comprehensive Multidisciplinary Care for the Patient with Cardiac Amyloidosis: A Report of the American College of Cardiology Solution Set Oversight Committee. J Am Coll Cardiol.

[9] Mallus MT, Rizzello V (2023). Treatment of Amyloidosis: Present and Future. Eur Heart J Suppl.

[10] Simões MV, Fernandes F, Marcondes-Braga FG, Scheinberg P, Correia EB, Rohde LEP (2021). Position Statement on Diagnosis and Treatment of Cardiac Amyloidosis - 2021. Arq Bras Cardiol.

[11] Rosenblum H, Castano A, Alvarez J, Goldsmith J, Helmke S, Maurer MS (2018). TTR (Transthyretin) Stabilizers are Associated with Improved Survival in Patients with TTR Cardiac Amyloidosis. Circ Heart Fail.

[12] Adams D, Gonzalez-Duarte A, O’Riordan WD, Yang CC, Ueda M, Kristen AV (2018). Patisiran, an RNAi Therapeutic, for Hereditary Transthyretin Amyloidosis. N Engl J Med.

[13] Wang J, Chen H, Tang Z, Zhang J, Xu Y, Wan K (2023). Tafamidis Treatment in Patients with Transthyretin Amyloid Cardiomyopathy: A Systematic Review and Meta-Analysis. EClinicalMedicine.

[14] Sukaina M, Rehman S, Waheed M, Shehryar M, Rasool R, Ahmed N (2023). Efficacy of Tafamidis in Transthyretin Amyloid Cardiomyopathy: A Systematic Review and Meta-Analysis. Ann Med Surg.

[15] Moher D, Liberati A, Tetzlaff J, Altman DG, PRISMA Group (2009). Preferred Reporting Items for Systematic Reviews and Meta-Analyses: The PRISMA Statement. BMJ.

[16] Higgins JPT, Thomas J, Chandler J, Cumpston M, Li T, Page MJ (2023). Cochrane Handbook for Systematic Reviews of Interventions version 6.4 (updated August 2023).

[17] (2024). Review Manager (RevMan) [Computer program]. Version 8.1.1.

[18] Sterne JAC, Savović J, Page MJ, Elbers RG, Blencowe NS, Boutron I (2019). RoB 2: A Revised Tool for Assessing Risk of Bias in Randomised Trials. BMJ.

[19] Schünemann H, Brożek J, Guyatt G, Oxman A (2013). GRADE Handbook [Internet].

[20] Judge DP, Kristen AV, Grogan M, Maurer MS, Falk RH, Hanna M (2020). Phase 3 Multicenter Study of Revusiran in Patients with Hereditary Transthyretin-Mediated (hATTR) Amyloidosis with Cardiomyopathy (ENDEAVOUR). Cardiovasc Drugs Ther.

[21] Gillmore JD, Judge DP, Cappelli F, Fontana M, Garcia-Pavia P, Gibbs S (2024). Efficacy and Safety of Acoramidis in Transthyretin Amyloid Cardiomyopathy. N Engl J Med.

[22] Maurer MS, Schwartz JH, Gundapaneni B, Elliott PM, Merlini G, Waddington-Cruz M (2018). Tafamidis Treatment for Patients with Transthyretin Amyloid Cardiomyopathy. N Engl J Med.

[23] Fontana M, Berk JL, Gillmore JD, Witteles RM, Grogan M, Drachman B (2025). Vutrisiran in Patients with Transthyretin Amyloidosis with Cardiomyopathy. N Engl J Med.

[24] Ioannou A, Fumagalli C, Razvi Y, Porcari A, Rauf MU, Martinez-Naharro A (2024). Prognostic Value of a 6-Minute Walk Test in Patients with Transthyretin Cardiac Amyloidosis. J Am Coll Cardiol.

[25] Spertus JA, Jones PG, Sandhu AT, Arnold SV (2020). Interpreting the Kansas City Cardiomyopathy Questionnaire in Clinical Trials and Clinical Care: JACC State-of-the-Art Review. J Am Coll Cardiol.

[26] Hood CJ, Hendren NS, Pedretti R, Roth LR, Saelices L, Grodin JL (2022). Update on Disease-Specific Biomarkers in Transthyretin Cardiac Amyloidosis. Curr Heart Fail Rep.

[27] Moody WE, Turvey-Haigh L, Knight D, Coats CJ, Cooper RM, Schofield R (2023). British Society of Echocardiography Guideline for the Transthoracic Echocardiographic Assessment of Cardiac Amyloidosis. Echo Res Pract.

[28] Benson MD, Waddington-Cruz M, Berk JL, Polydefkis M, Dyck PJ, Wang AK (2018). Inotersen Treatment for Patients with Hereditary Transthyretin Amyloidosis. N Engl J Med.

[29] Maurer MS, Kale P, Fontana M, Berk JL, Grogan M, Gustafsson F (2023). Patisiran Treatment in Patients with Transthyretin Cardiac Amyloidosis. N Engl J Med.

