# Supplementary material for: Terapias Modificadoras de Doença para a Cardiomiopatia por Amiloidose por Transtirretina: Uma Revisão Sistemática e Metanálise
Source: Arq Bras Cardiol. 2025 Sep 10;122(8):e20240830. [Article in Portuguese] doi: 10.36660/abc.20240830 (PMC12677762; doi:10.36660/abc.20240830)
Supplement: Supplementary file 1 [file 0066-782X-abc-122-08-e20240830-Suppl01.pdf]

Apêndice Suplementar

Protocolo:

1. Condição ou domínio em estudo

A amiloidose cardíaca é uma cardiomiopatia infiltrativa e restritiva que leva à insuficiência cardíaca e redução da qualidade de vida.[1] A doença é dividida em dois subtipos: amiloidose cardíaca de cadeia leve de imunoglobulina (AL) e amiloidose cardíaca transtirretina (ATTR).[2] A amiloidose cardíaca ATTR é caracterizada por um defeito na formação da proteína transtirretina pelo fígado, o que pode levar à formação excessiva de depósitos de fibrilas amiloides no miocárdio.[3] É uma condição que é cada vez mais diagnosticada devido ao surgimento de diagnósticos de imagem não invasivos e à crescente conscientização sobre sua prevalência, o que leva a um diagnóstico mais precoce e frequente.[4] Nesse contexto, as terapias modificadoras da doença têm o potencial de prevenir a progressão da doença.[5] Nossa metanálise avalia especificamente essas terapias na ATTR-CM.

2. Participantes/população

Incluiremos ensaios clínicos randomizados (ECRs) comparando o tratamento farmacológico modificador da doença com amiloidose cardíaca ATTR versus placebo em pacientes com diagnóstico de amiloidose, relatando os desfechos clínicos de interesse. Excluiremos estudos com populações de pacientes sobrepostas, bem como estudos sem um grupo de controle.

3. Intervenção(ões), exposição(ões)

Este estudo teve como objetivo avaliar se terapias farmacológicas destinadas a prevenir a progressão da amiloidose cardíaca por transtirretina são eficazes em

comparação ao placebo. Foram incluídos apenas ensaios clínicos randomizados (ECRs) que investigassem a eficácia de agentes modificadores da doença em pacientes com diagnóstico confirmado de amiloidose ATTR e evidência de comprometimento cardíaco, desde que relatassem pelo menos um desfecho clínico relevante. Os critérios de exclusão abrangeram estudos sem grupo controle com placebo, populações sobrepostas, resumos de congressos, editoriais, revisões, comentários e relatos de casos.

4. Comparador/controle

O placebo foi utilizado como intervenção de controle em todos os ensaios incluídos. Os grupos comparadores receberam o tratamento padrão, sem terapias farmacológicas modificadoras da doença para amiloidose cardíaca por transtirretina, permitindo a avaliação isolada do efeito da intervenção estudada.

5. Tipos de estudo a serem incluídos

Somente ensaios clínicos randomizados serão incluídos.

6. Contexto

Considerando o surgimento de diversas novas terapias farmacológicas para a amiloidose cardíaca por transtirretina (ATTR), este estudo foi desenvolvido com o objetivo de avaliar se essas intervenções modificam os desfechos cardíacos em comparação ao placebo, incluindo análises por subgrupos terapêuticos. Serão incluídos apenas estudos que reportem ao menos um dos desfechos de interesse.

7. Principais desfechos

Os desfechos primários considerados serão mortalidade por todas as causas, mortalidade cardiovascular e hospitalizações por todas as causas, cardiovasculares e relacionadas à insuficiência cardíaca. Adicionalmente, serão avaliadas medidas de efeito, incluindo risco relativo, diferença absoluta de risco e número necessário para tratar (NNT), quando disponíveis, em comparação ao placebo.

8. Desfecho(s) adicional(ais)

Em relação aos resultados secundários, analisaremos medidas ecocardiográficas, biomarcadores cardíacos e avaliaremos riscos relativos, diferença de risco e/ou número necessário para tratar quando disponível em comparação ao placebo.

9. Extração de dados (seleção e codificação)

Todos os artigos identificados com a estratégia de busca no banco de dados eletrônico serão exportados para o software Zotero, e duplicatas serão removidas. A leitura inicial de títulos e resumos será realizada de forma independente por dois revisores que não conhecem as seleções um do outro. Desacordos serão resolvidos por meio de discussão e, se necessário, consulta com um terceiro revisor para chegar a um consenso. Todos os artigos potencialmente relevantes serão analisados em detalhes no texto completo. As decisões serão registradas usando uma ferramenta de uma planilha Excel.

Os dados a serem extraídos dos estudos incluídos compreenderão: nome do primeiro autor, título, desenho do estudo, ano de publicação, país de realização, tempo de seguimento e tamanho da amostra. As características basais dos participantes serão descritas por número de pacientes (média), idade (média), sexo (feminino/masculino), raça (branca/negra/asiática) e, em relação às variáveis clínicas, serão coletados a fração de ejeção do ventrículo esquerdo (FEVE), classe funcional da insuficiência cardíaca

(NYHA), genótipo ATTR (mutado ou tipo selvagem), índice de massa corporal (IMC) e tempo de acompanhamento (em meses).

Em relação aos desfechos, serão extraídas as taxas de mortalidade e hospitalizações por todas as causas, hospitalizações cardíacas e hospitalizações por insuficiência cardíaca, com suas respectivas razões de risco e intervalos de confiança de 95%, provenientes de análises univariadas e multivariadas, quando disponíveis, incluindo as variáveis ajustadas.

Em caso de indisponibilidade de dados relevantes, os autores dos estudos serão contatados para obtenção de informações adicionais e/ou esclarecimentos.

10. Avaliação de risco de viés (qualidade)

O risco de viés será avaliado de forma independente por dois revisores. Quaisquer divergências serão resolvidas por consenso e, se necessário, com a participação de um terceiro revisor.

A qualidade metodológica dos estudos será analisada por meio da ferramenta *Risk of Bias 2* (RoB 2), desenvolvida pela *Cochrane Collaboration*, para avaliação do risco de viés em ensaios clínicos randomizados.

11. Estratégia para síntese de dados

A revisão sistemática e a meta-análise serão realizadas de acordo com as recomendações da *Cochrane Collaboration* e as diretrizes da declaração PRISMA (*Preferred Reporting Items for Systematic Reviews and Meta-Analyses*). Os dados dos estudos individuais serão extraídos com base nas taxas de risco. Os efeitos do tratamento para desfechos binários serão comparados por meio do risco relativo (RR), e para

desfechos contínuos, pela diferença de médias (DM), ambos com intervalos de confiança de 95%.

A heterogeneidade será avaliada por meio da estatística I^2 de Higgins e explorada por análises de subgrupos. A estimativa de Tau^2 será obtida pelo método de DerSimonian e Laird, com uso de modelos de efeitos aleatórios.

Todas as análises estatísticas serão conduzidas no software RevMan, versão 5.4 (Nordic Cochrane Centre, *The Cochrane Collaboration*, Copenhague, Dinamarca).

12. Análise de subgrupos ou subconjuntos

**Tabela S1. Estratégia de Busca**	
**Embase**	(amyloidosis OR amyloid) AND (ATTR OR transthyretin) AND (vutrisiran OR revusiran OR patisiran OR tafamidis OR inotersen OR doxycycline OR diflunisal OR tolcapone OR "ursodeoxycholic acid" OR UDCA OR tauroursodeoxycholic OR TUDCA OR "AG-10" OR AG10 OR acoramidis) AND ('randomized controlled trial'/exp OR 'controlled clinical trial'/exp OR randomized:ti,ab OR placebo:ti,ab OR 'drug therapy':lnk OR randomly:ti,ab OR trial:ti,ab OR groups:ti,ab)
**Cochrane**	(amyloidosis OR amyloid) AND (ATTR OR transthyretin) AND (vutrisiran OR revusiran OR patisiran OR tafamidis OR inotersen OR doxycycline OR diflunisal OR tolcapone OR "ursodeoxycholic acid" OR UDCA OR tauroursodeoxycholic OR TUDCA OR "AG-10" OR AG10 OR acoramidis) AND ("randomized controlled trial" OR "controlled clinical trial" OR randomized OR placebo OR "drug therapy" OR randomly OR trial OR groups)
**Pubmed**	(amyloidosis OR amyloid) AND (ATTR OR transthyretin) AND (vutrisiran OR revusiran OR patisiran OR tafamidis OR inotersen OR doxycycline OR diflunisal OR tolcapone OR "ursodeoxycholic acid" OR UDCA OR tauroursodeoxycholic OR TUDCA OR "AG-10" OR AG10 OR acoramidis) AND ("randomized controlled trial" [pt] OR "controlled clinical trial" [pt] OR randomized [tiab] OR placebo [tiab] OR "drug therapy" [sh] OR randomly [tiab] OR trial [tiab] OR groups [tiab])

Caso haja um número suficiente de estudos, serão realizadas análises de subgrupos com base nos desfechos acima descritos.

Tabela S2. Desfechos extraídos de acordo com os ECRs

OUTCOMES	ATTR-ACT Estabilizador	ATTRibute-CM Estabilizador	APOLLO Silenciador	NEURO-TTR Silenciador	ENDEAVOUR Silenciador	APOLLO-B Silenciador	HELIOS-B Silenciador
**Mortalidade (todas as causas)**	*	*	†		*	*	*
**Mortalidade Cardíaca**			†		*	*	
**Hospitalizações (todas as causas)**	†	*	††		*	*	
**Hospitalizações Cardíacas**	*		††		*		
**Hospitalizações IC**	*				*	*	
**TC6M**	*	*			*	*	*
**KCCQ-OS**	*	*				*	*
**NT-proBNP Sérico**	†§	*	†			*	*
**TTR Sérica**		*				*	*
**SLG de VE**	*		†	*		*	*
**Massa de VE**			†	*	§	*	
**Espessura de Parede de VE**	*		†	*	§	*	
**FEVE**	*		†	*	§		

*: dados do ECR original[6-12]; †: dados de análise post-hoc[13-16]; §: dados imputados; ††: dados não utilizados, pois estavam relacionados à polineuropatia; TC6M: teste de caminhada de 6 minutos; SLG: strain longitudinal global; IC: insuficiência cardíaca; KCCQ-OS: escore geral do questionário de cardiomiopatia de Kansas City; VE: ventrículo esquerdo; FEVE: fração de ejeção do ventrículo esquerdo; NT pro-BNP: pró-hormônio N-terminal do peptídeo natriurético cerebral; TTR: transtirretina.

Tabela S3. Avaliação da classificação de recomendações, avaliação, desenvolvimento e avaliação (GRADE)

Estabilizadores de TTR ou silenciadores de TTR comparados ao placebo para amiloidose cardíaca											
**Avaliação da Certeza**							**Resumo dos Achados**				
							**Taxa de Eventos do Estudo (%)**			**Efeitos Absolutos Potenciais**	
**Participantes (Estudos)**	**Risco de Viés**	**Inconsistência**	**Evidência Indireta**	**Imprecisão**	**Viés de Publicação**	**Certeza geral da evidência**	**Com placebo**	**Estabilizadores ou Silenciadores de TTR**	**Efeito Relativo (IC 95%)**	**Risco com placebo**	**Diferença de Risco com Estabilizadores ou Silenciadores TTR**
**Mortalidade (todas as causas) – Estabilizadores de TTR**											
1052 (2 ECRs)	não grave	não grave	não grave	não grave	não detectado	⊕⊕⊕⊕ Alta	128/379 (33.8%)	157/673 (23.3%)	**RR 0.71** (0.59 a 0.87)	128/379 (33.8%)	**Redução de 98 casos a cada 1.000** (de 138 a menos até 44 a menos)
**Mortalidade (todas as causas) - Silenciadores de TTR**											
1345 (4 ECRs)	grave[a]	muito grave[b]	não grave	grave[c]	não detectado	⊕◯◯◯ Muito Baixa[a,b,c]	101/608 (16.6%)	87/737 (11.8%)	**RR 0.79** (0.37 a 1.68)	101/608 (16.6%)	**Redução de 35 casos a cada 1.000** (de 105 a menos ate 113 a mais)
**Mortalidade Cardiovascular - Estabilizadores de TTR: Desfecho não disponível**											
**Mortalidade Cardiovascular – Silenciadores de TTR**											
691 (3 ECRs)	não grave	não grave	não grave	muito grave[c]	não detectado	⊕⊕◯◯ Baixa[c]	6/280 (2.1%)	23/411 (5.6%)	**RR 1.87** (0.64 a 5.44)	6/280 (2.1%)	**Aumento de 19 casos a cada 1.000** (de 8 a menos ate 95 a mais)
**Hospitalizações (todas as causas) - Estabilizadores de TTR**											

Estabilizadores de TTR ou silenciadores de TTR comparados ao placebo para amiloidose cardíaca											
Avaliação da Certeza							Resumo dos Achados				
1073<br>(2 ECRs)	não grave	não grave	não grave	não grave	não detectado	⊕⊕⊕⊕<br>Alta	264/388<br>(68.0%)	402/685<br>(58.7%)	**RR 0.81**<br>(0.73 a 0.89)	264/388<br>(68.0%)	**Redução de 129 caso a cada 1.000**<br>(de 184 a menos ate 75 a menos)
**Hospitalizações (todas as causas) – Silenciadores de TTR**											
565<br>(2 ECRs)	grave[a]	grave[b]	não grave	muito grave[c]	não detectado	⊕◯◯◯<br>Muito Baixa[a,b,c]	89/244<br>(36.5%)	132/321<br>(41.1%)	**RR 1.11**<br>(0.84 a 1.47)	89/244<br>(36.5%)	**Aumento de 40 casos a cada 1.000**<br>(de 58 a menos ate 171 a mais)
**Hospitalizações Cardíacas - Estabilizadores de TTR**											
441<br>(1 ECR)	não grave	não grave	não grave	muito grave[c]	não detectado	⊕⊕◯◯<br>Baixa[c]	107/177<br>(60.5%)	138/264<br>(52.3%)	**RR 0.86**<br>(0.73 a 1.02)	107/177<br>(60.5%)	**Redução de 85 casos a cada 1.000**<br>(de 163 a menos 12 a mais)
**Hospitalizações Cardíacas - Silenciadores de TTR**											
206<br>(1 ECR)	Muito grave[a]	não grave	não grave	muito grave[c]	não detectado	⊕◯◯◯<br>Muito Baixa[a,c]	21/66<br>(31.8%)	49/140<br>(35.0%)	**RR 1.10**<br>(0.72 a 1.67)	21/66<br>(31.8%)	**Aumento de 32 casos a cada 1.000**<br>(de 89 a menos ate 213 a mais)
**Hospitalizações IC – Estabilizadores de TTR**											
441<br>(1 ECR)	não grave	não grave	não grave	muito grave[c]	não detectado	⊕⊕◯◯<br>Baixa[c]	60/177<br>(33.9%)	76/264<br>(28.8%)	**RR 0.85**<br>(0.64 a 1.12)	60/177<br>(33.9%)	**Redução de 51 casos a cada 1.000**<br>(de 122 a menos até 41 a mais)

Estabilizadores de TTR ou silenciadores de TTR comparados ao placebo para amiloidose cardíaca											
**Avaliação da Certeza**							**Resumo dos Achados**				
**Hospitalizações IC – Silenciadores de TTR**											
565 (2 RCTs)	grave[a]	não grave	não grave	muito grave[c]	não detectado	⊕◯◯◯ Muito baixa[a,c]	17/244 (7.0%)	45/321 (14.0%)	**RR 1.40** (0.84 a 2.34)	17/244 (7.0%)	**Aumento de 28 casos a cada 1.000** (de 11 a menos até 93 a mais)
**TC6M - Estabilizadores de TTR**											
795 (2 RCTs)	não grave	não grave	não grave	grave[d]	não detectado	⊕⊕⊕◯ Moderada[d]	256	539	-	-	DM **57.16 maior** (21.92 maior até 92.4 maior)
**TC6M - Silenciadores de TTR**											
1037 (3 RCTs)	grave[a]	grave[b]	não grave	grave[d]	não detectado	⊕◯◯◯ Muito Baixa[a,b,d]	496	541	-	-	DM **18.02 maior** (4.22 maior até 31.82 maior)
**KCCQ-OS - Estabilizadores de TTR**											
860 (2 RCTs)	não grave	não grave	não grave	não grave	não detectado	⊕⊕⊕⊕ Alta	285	575	-	-	DM **11.55 maior** (7.89 maior até 15.22 maior)
**KCCQ-OS - Silenciadores de TTR**											
938 (2 ECRs)	não grave	não grave	não grave	grave[d]	não detectado	⊕⊕⊕◯ Moderada[d]	462	476	-	-	DM **4.82 maior** (2.39 maior até 7.25 maior)

Estabilizadores de TTR ou silenciadores de TTR comparados ao placebo para amiloidose cardíaca											
Avaliação da Certeza							Resumo dos Achados				
**NT pro-BNP Sérico – Estabilizadores de TTR**											
845<br>(2 ECRs)	não grave	não grave	não grave	não grave	não detectado	⊕⊕⊕⊕<br>Alta	278	567	-	-	DM **1.42 menor**<br>(1.67 menor até 1.16 menor)
**NT pro-BNP Sérico – Silenciadores de TTR**											
1106<br>(3 ECRs)	não grave	não grave	não grave	grave[d]	não detectado	⊕⊕⊕◯<br>Moderada[d]	525	581	-	-	DM **0.42 menor**<br>(0.7 menor até 0.15 menor)
**TTR sérica - Estabilizadores de TTR**											
594<br>(1 ECR)	não grave	não grave	não grave	grave[e]	não detectado	⊕⊕⊕◯<br>Moderada[e]	197	397	-	-	DM **47.76 menor**<br>(49.02 menor até 46.5 menor)
**TTR sérica - Silenciadores de TTR**											
798<br>(2 ECRs)	não grave	não grave	não grave	não grave	não detectado	⊕⊕⊕⊕<br>Alta	295	294	-	-	DM **81.62 menor**<br>(86.44 menor até 76.8 menor)
**SLG de VE - Estabilizadores de TTR**											
441<br>(1 ECR)	não grave	não grave	não grave	muito grave[c]	não detectado	⊕⊕◯◯<br>Baixa[c]	177	264	-	-	DM **0.7 menor**<br>(1.55 menor até 0.15 maior)
**SLG de VE - Silenciadores de TTR**											

Estabilizadores de TTR ou silenciadores de TTR comparados ao placebo para amiloidose cardíaca											
Avaliação da Certeza							Resumo dos Achados				
1177<br>(4 ECRs)	não grave	grave[f]	não grave	grave[d]	não detectado	⊕⊕◯◯<br>Baixa[d,f]	547	630	-	-	DM **0.85 menor**<br>(1.41 menor até 0.3 menor)
**Massa de VE - Estabilizadores de TTR - Desfecho não disponível**											
**Massa de VE – Silenciadores de TTR**											
655<br>(4 ECRs)	não grave	não grave	não grave	grave[d]	não detectado	⊕⊕⊕◯<br>Moderada[d]	267	388	-	-	DM **9.74 menor**<br>(17.07 menor até 2.4 menor)
**Espessura de Parede de VE – Estabilizadores de TTR**											
441<br>(1 ECR)	não grave	não grave	não grave	muito grave[c]	não detectado	⊕⊕◯◯<br>Baixa[c]	177	264	-	-	DM **0.44 menor**<br>(1.25 menor até 0.37 menor)
**Espessura de Parede de VE - Silenciadores de TTR**											
657<br>(4 ECRs)	muito grave[a]	muito grave[b]	não grave	grave[c]	não detectado	⊕◯◯◯<br>Muito Baixa[a,b,c]	268	389	-	-	DM **0.28 menor**<br>(0.67 menor até 0.11 maior)
**FEVE - Estabilizadores de TTR**											
441<br>(1 ECR)	não grave	não grave	não grave	muito grave[c]	não detectado	⊕⊕◯◯<br>Baixa[c]	177	264	-	-	DM **1.52 maior**<br>(1.2 menor até 4.24 maior)
**FEVE - Silenciadores de TTR**											

**Estabilizadores de TTR ou silenciadores de TTR comparados ao placebo para amiloidose cardíaca**											
**Avaliação da Certeza**							**Resumo dos Achados**				
318 (3 ECRs)	não grave	não grave	não grave	muito grave[c]	não detectado	⊕⊕◯◯ Baixa[c]	103	215	-	-	DM **0.99 menor** (3.05 menor até 1.06 maior)

[a] Nesta classe terapêutica, a certeza da evidência foi rebaixada devido ao alto risco de viés causado pelo estudo ENDEAVOUR, que foi descontinuado precocemente.
[b] Há inconsistência entre os estudos, principalmente devido ao estudo ENDEAVOUR.
[c] Os extremos do intervalo de confiança de 95% podem levar a decisões clínicas diferentes.
[d] O resultado pode não oferecer um benefício clínico significativo ao paciente.
[e] O resultado do estudo é incerto para informar decisões clínicas.
[f] Nesta classe terapêutica, a inconsistência foi reduzida devido ao estudo NEURO-TTR.

IC: intervalo de confiança; DM: diferença de médias; RR: risco relativo

Tabela S4. Mudança anual ajustada por placebo acompanhada na capacidade funcional (TC6M) e qualidade de vida (KCCQ-OS) por 1 ano[a]

TC6M	Classe	Medicação	Intervenção (m)	Placebo (m)	Melhora (%)	Seguimento (meses)	Melhora Anual (m)	Melhora Anual (%)
**ATTR-ACT**	Estabilizador	Tafamidis	-54.89	-130.5	58	30	30.24	23
**ATTRibute-CM**	Estabilizador	Acoramidis	-64.56	-104.2	38	30	15.85	15
**APOLLO-B**	Silenciador	Patisiran	-11.36	-29.51	61	12	18.15	61
**HELIOS-B**	Silenciador	Vutrisiran	-59.7	-91.8	35	30	12.84	14
**KCCQ-OS**	**Classe**	**Medicação**	**Intervenção (pontos)**	**Placebo (pontos)**	**Melhora (%)**	**Seguimento (meses)**	**Melhora Anual (m)**	**Melhora Anual (%)**
**ATTR-ACT**	Estabilizador	Tafamidis	-7.17	-20.84	65	30	5.46	26
**ATTRibute-CM**	Estabilizador	Acoramidis	-11.5	-21.4	46	30	3.96	18
**APOLLO-B**	Silenciador	Patisiran	0.3	-3.4	108	12	3.7	108
**HELIOS-B**	Silenciador	Vutrisiran	-10.8	-19.5	44	30	3.48	18

[a]Estudo aENDEAVOUR não foi analisado devido ao acompanhamento menor de 1 ano; m: metros.

Figura S1. Avaliação de qualidade RoB 2

Estudo	Domínios do risco de viés					
	D1	D2	D3	D4	D5	Overall
APPOLO (Patisiran – Mortalidade todas as causas)	+	+	+	+	+	+
ATTR ACT (Tafamidis – Mortalidade todas as causas)	+	+	+	+	+	+
ENDEAVOUR (Revusiran – Mortalidade todas as causas)	+	+	+	-	+	-
APOLLO B (Patisiran – Mortalidade todas as causas)	+	+	+	+	+	+
ATTRIBUTE (Acoramidis – Mortalidade todas as causas)	+	+	+	+	+	+
HELIOS-B (Vutrisiran – Mortalidade todas as causas)	+	+	+	+	+	+
APOLLO (Patisiran – Mortalidade cardíaca)	+	+	+	+	+	+
ENDEAVOUR (Revusiran – Mortalidade cardíaca)	+	+	+	-	+	-
APOLLO B (Patisiran – Mortalidade cardíaca)	+	+	+	+	+	+
ENDEAVOUR (Revusiran – Hospitalizações todas as causas)	+	+	+	-	+	-
APOLLO B (Patisiran – Hospitalizações todas as causas)	+	+	+	+	+	+
ATTRIBUTE (Acoramidis – Hospitalizações todas as causas)	+	+	+	+	+	+
ATTR ACT (Tafamidis – Hospitalizações Cardíacas)	+	+	+	+	+	+
ENDEAVOUR (Revusiran – Hospitalizações Cardíacas)	+	+	+	-	+	-
ATTR ACT (Tafamidis – Hospitalizações IC)	+	+	+	+	+	+
ENDEAVOUR (Revusiran – Hospitalizações IC)	+	+	+	-	+	-
APOLLO B (Patisiran – Hospitalizações IC)	+	+	+	+	+	+
ATTR ACT (Patisiran – TC6M)	+	+	+	+	+	+
ENDEAVOUR (Revusiran – TC6M)	+	+	X	-	+	X
APOLLO B (Patisiran – TC6M)	+	+	+	+	+	+
ATTRIBUTE (Acoramidis – TC6M)	+	+	+	+	+	+
HELIOS-B (Vutrisiran – TC6M)	+	+	+	+	+	+
ATTR-ACT (Tafamidis – KCCQ-OS)	+	+	-	+	+	-
APOLLO B (Patisiran – KCCQ-OS)	+	+	+	+	+	+
ATTRIBUTE (Acoramidis – KCCQ-OS)	+	+	+	+	+	+
HELIOS-B (Vutrisiran – KCCQ-OS)	+	+	+	+	+	+
APOLLO (Patisiran – NT pro-BNP)	+	+	+	+	+	+
ATTR-ACT (Tafamidis – NT pro-BNP)	+	+	+	+	+	+
APOLLO B (Patisiran – NT pro-BNP)	+	+	+	+	+	+
ATTRIBUTE (Acoramidis – NT pro-BNP)	+	+	+	+	+	+
HELIOS-B (Vutrisiran – NT pro-BNP)	+	+	+	+	+	+

APOLLO (Patisiran – TTR Sérica)	+	+	+	+	+	+
NEURO TTR (Inotersen – TTR Sérica)	+	+	+	+	+	+
APOLLO B (Patisiran – TTR Sérica)	+	+	+	+	+	+
ATTRIBUTE (Acoramidis – TTR Sérica)	+	+	+	+	+	+
HELIOS-B (Vutrisiran – TTR Sérica)	+	+	+	+	+	+
APOLLO (Patisiran – GLS de VE)	+	+	+	+	+	+
NEURO TTR (Inotersen – GLS de VE)	+	+	+	+	+	+
ATTR ACT (Tafamidis – GLS de VE)	+	+	+	+	+	+
APOLLO B (Patisiran – GLS de VE)	+	+	+	+	+	+
HELIOS-B (Vutrisiran – GLS de VE)	+	+	+	+	+	+
APOLLO (Patisiran – Massa de VE)	+	+	+	+	+	+
NEURO TTR (Inotersen – Massa de VE)	+	+	+	+	+	+
ENDEAVOUR (Revusiran – Massa de VE)	+	+	X	-	+	X
APOLLO B (Patisiran – Massa de VE)	+	+	+	+	+	+
APOLLO (Patisiran – Espessura de parede de VE)	+	+	+	+	+	+
NEURO TTR (Inotersen – Espessura de parede de VE)	+	+	+	+	+	+
ATTR ACT (Tafamidis – Espessura de parede de VE)	+	+	+	+	+	+
ENDEAVOUR (Revusiran – Espessura de parede de VE)	+	+	X	-	+	X
APOLLO B (Patisiran – Espessura de parede de VE)	+	+	+	+	+	+
APOLLO (Patisiran – FEVE)	+	+	+	+	+	+
NEURO TTR (Inotersen – FEVE)	+	+	+	+	+	+
ATTR ACT (Tafamidis – FEVE)	+	+	+	+	+	+
ENDEAVOUR (Revusiran – FEVE)	+	+	X	-	+	X

Domínios:

D1: Viés decorrente do processo de randomização
D2: Viés devido a desvios da intervenção pretendida
D3: Viés devido a dados de desfecho ausentes
D4: Viés na mensuração do desfecho
D5: Viés na seleção do resultado reportado

Julgamento:

X Alto
- Algumas preocupações
+ Baixo

O risco de viés foi analisado com base nos desfechos. O Domínio 3 foi considerado de alto risco de viés ou apresentou algumas preocupações devido a dados ausentes. O Domínio 4 apresentou algumas preocupações relacionadas à mensuração dos desfechos, uma vez que o estudo ENDEAVOUR foi descontinuado precocemente.

Figura S2. TTR sérica para terapias modificadoras da doença ATTR-CM *versus* placebo

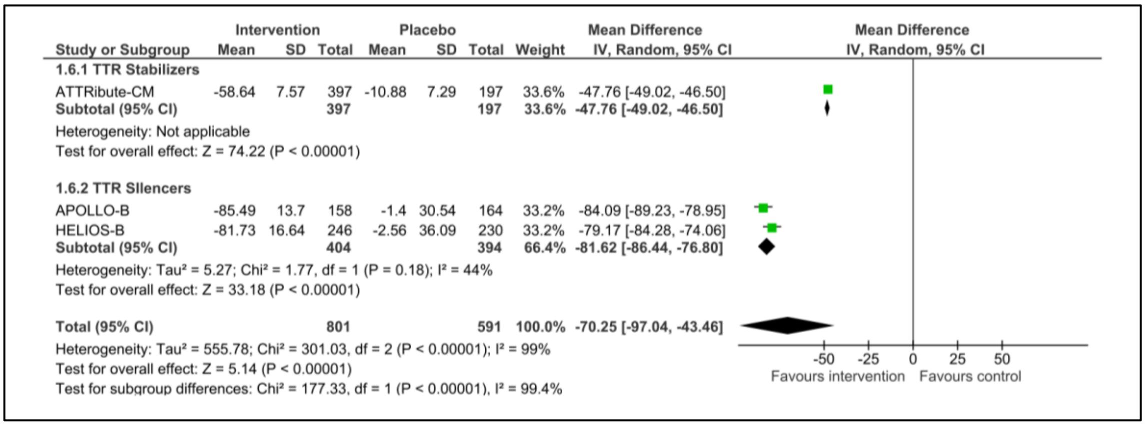

Chi^2: Chi-square; CI: Confidence Interval; df: degrees of freedom; I^2: I-squared; IV: Inverse Variance; p: p-value; Tau: Kendall's Tau; TTR: transthyretin.

Figura S3A. Avaliação ecocardiográfica do SLG do VE para terapias modificadoras da doença CA-TTR *versus* placebo

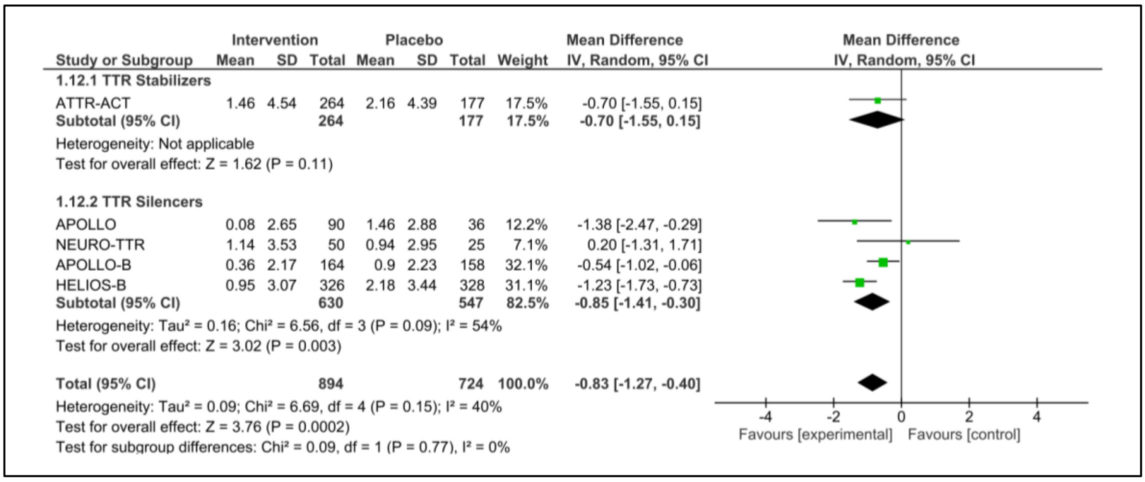

Chi²: Chi-square; CI: Confidence Interval; df: degrees of freedom; GLS: global longitudinal strain; I²: I-squared; IV: Inverse Variance; LV: left ventricular; p: p-value; Tau: Kendall's Tau; TTR: transthyretin.

Figura S3B. Avaliação ecocardiográfica da massa do VE para terapias modificadoras da doença CA-TTR *versus* placebo

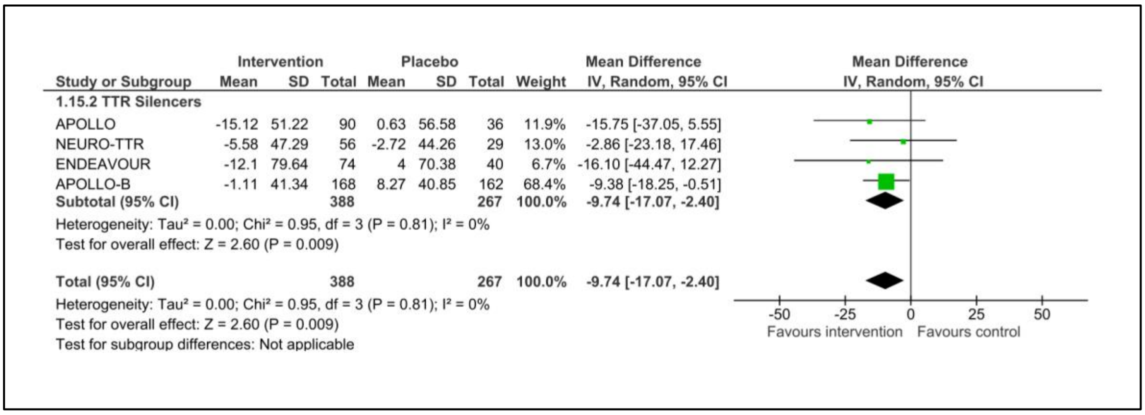

Chi²: Chi-square; CI: Confidence Interval; df: degrees of freedom; I²: I-squared; IV: Inverse Variance; LV: left ventricular; p: p-value; Tau: Kendall's Tau; TTR: transthyretin.

Figura S3C. Avaliação ecocardiográfica da espessura de parede de VE para terapias modificadoras da doença CA-TTR *versus* placebo

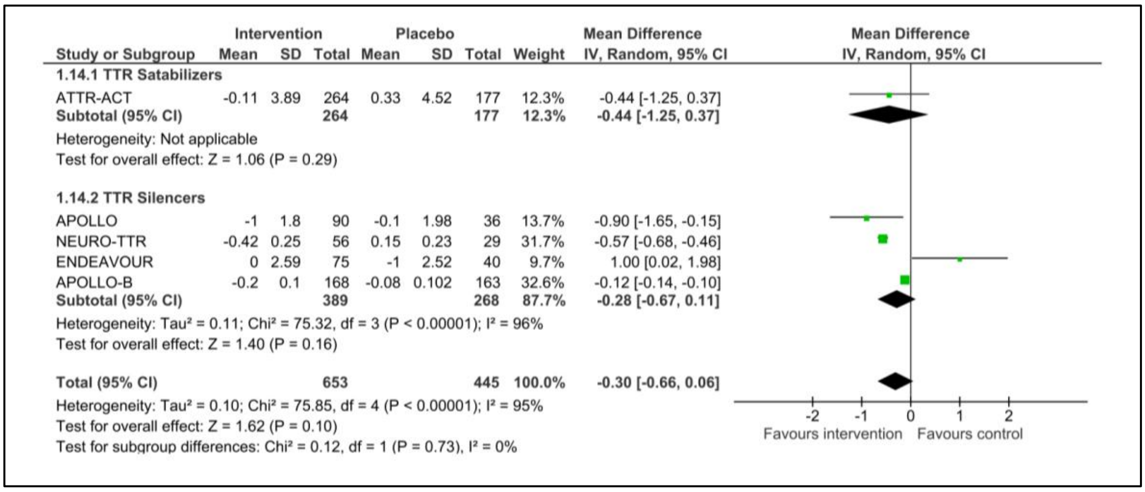

Chi^2: Chi-square; CI: Confidence Interval; df: degrees of freedom; I^2: I-squared; IV: Inverse Variance; LV: left ventricular; p: p-value; Tau: Kendall's Tau; TTR: transthyretin.

Figura SD3. Avaliação ecocardiográfica da FEVE para terapias modificadoras da doença CA-TTR *versus* placebo

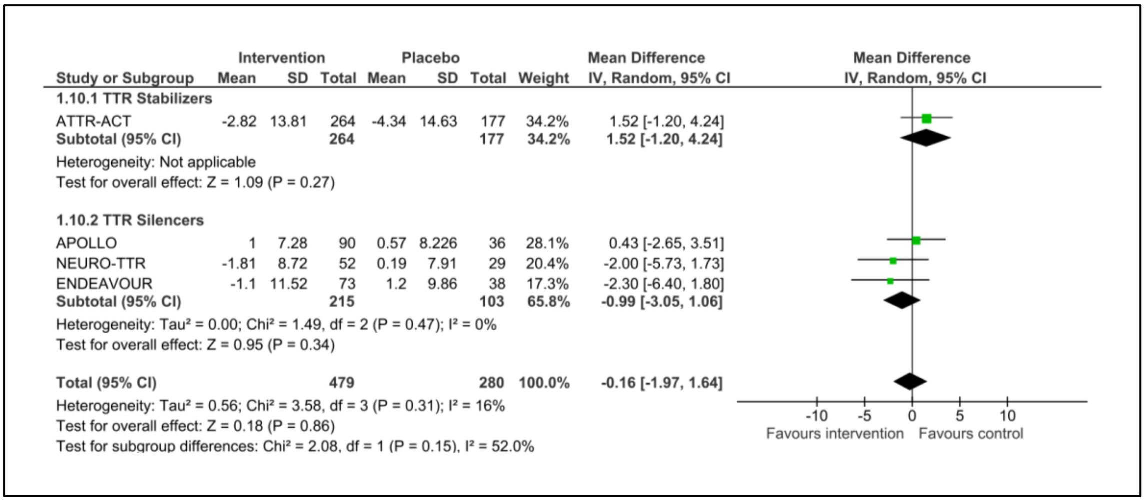

Chi^2: Chi-square; CI: Confidence Interval; df: degrees of freedom; I^2: I-squared; IV: Inverse Variance; LVEF: left ventricular ejection fraction; p: p-value; Tau: Kendall's Tau; TTR: transthyretin.

Figura S4A. Mortalidade por todas as causas para terapias modificadoras da doença CA-TTR *versus* placebo

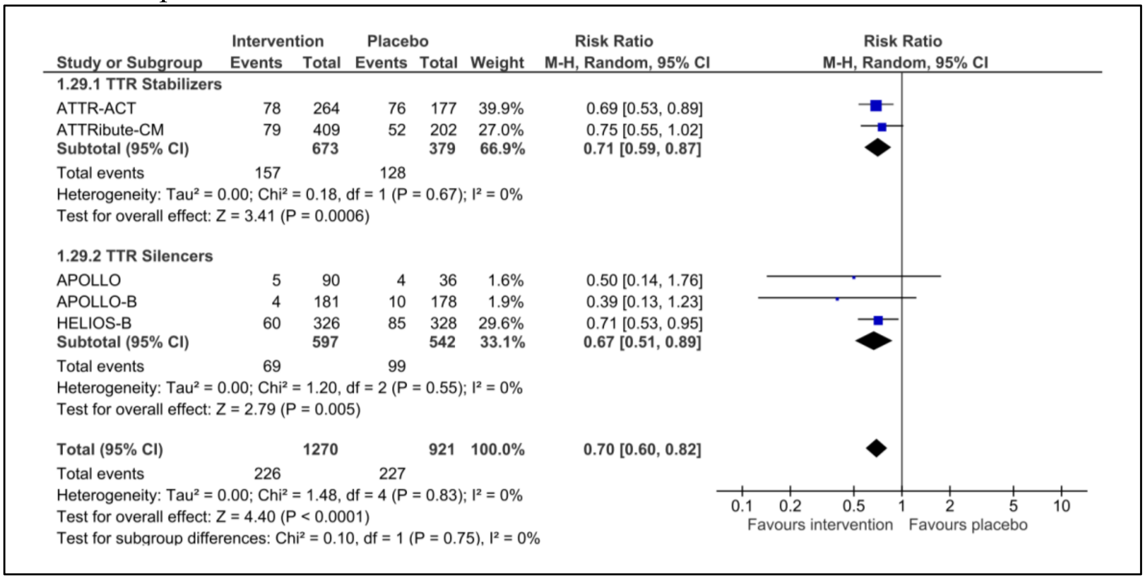

Esta análise foi conduzida sem incorporar dados do estudo ENDEAVOUR. *Chi²: Chi-square; CI: Confidence Interval; df: degrees of freedom; I²: I-squared; M-H: Mantel-Haenszel; p: p-value; Tau: Kendall's Tau; TTR: transthyretin.*

Figura S4B. Avaliação ecocardiográfica de espessura de VE para terapias modificadoras da doença CA-TTR *versus* placebo

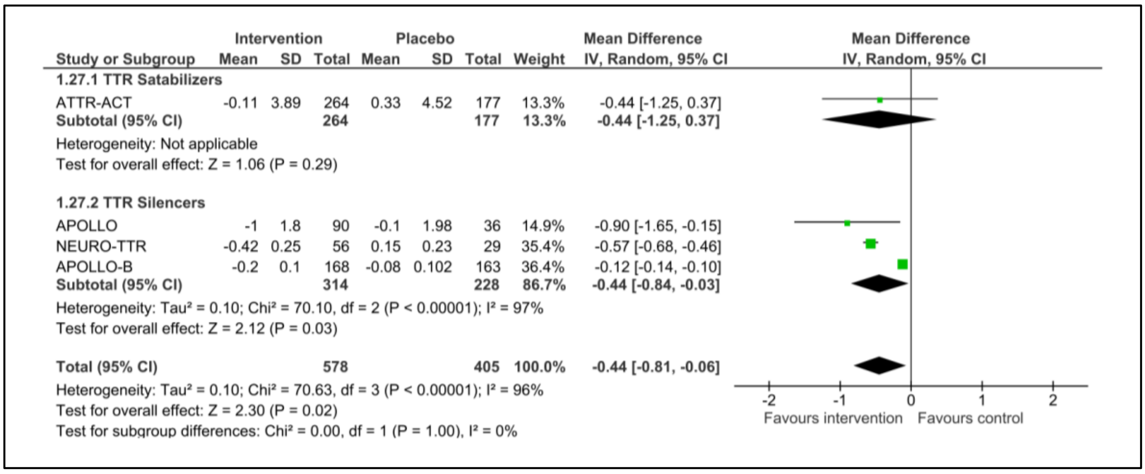

Esta análise foi conduzida sem incorporar dados do estudo ENDEAVOUR. *Chi²: Chi-square; CI: Confidence Interval; df: degrees of freedom; I²: I-squared; IV: Inverse Variance; LV: left ventricular; p: p-value; Tau: Kendall's Tau; TTR: transthyretin.*

Figure S5A. Mortalidade por todas as causas para terapias modificadoras da doença CA-TTR *versus* placebo

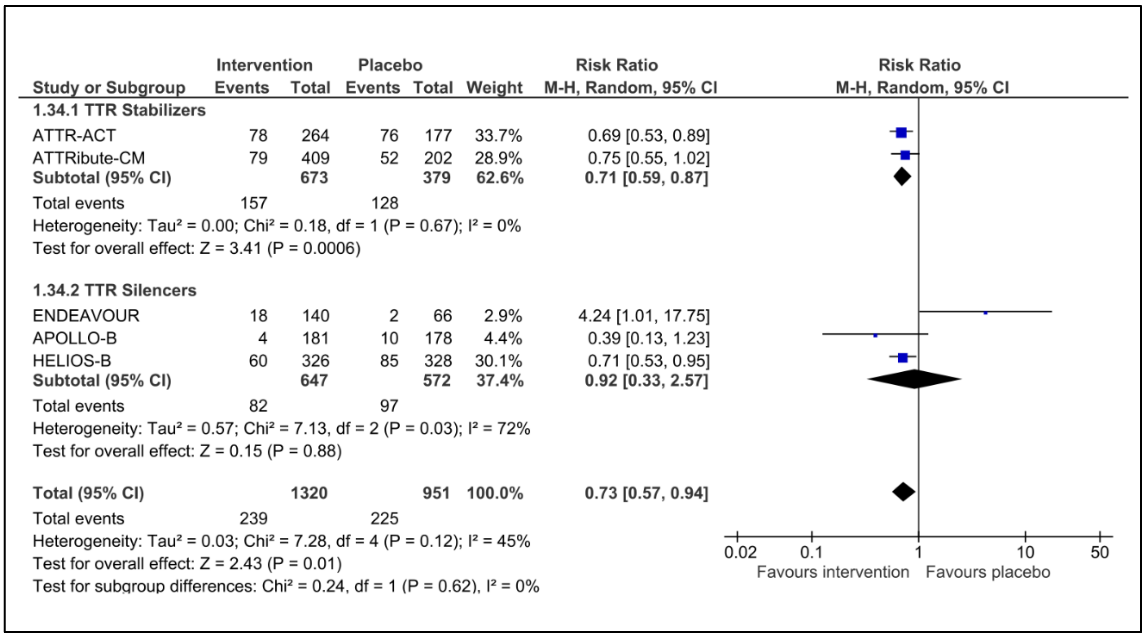

Esta análise foi conduzida sem incorporar dados do estudo APOLLO. *Chi²: Chi-square; CI: Confidence Interval; df: degrees of freedom; I²: I-squared; M-H: Mantel-Haenszel; p: p-value; Tau: Kendall's Tau; TTR: transthyretin.*

Figure S5B. NT pro-BNP sérico para terapias modificadoras da doença ATTR-CM *versus* placebo

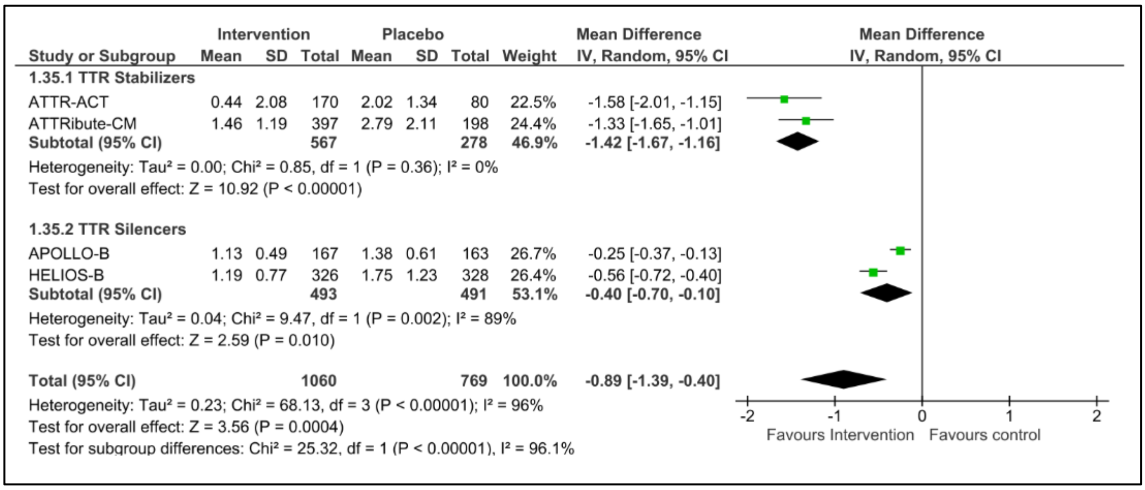

Esta análise foi conduzida sem incorporar dados do estudo APOLLO. *Chi²: Chi-square; CI: Confidence Interval; df: degrees of freedom; I²: I-squared; IV: Inverse Variance; p: p-value; NT pro-BNP: N-terminal prohormone of brain natriuretic peptide; Tau: Kendall's Tau; TTR: transthyretin.*

References:

1. Simões MV, Fernandes F, Marcondes-Braga FG, Scheinberg P, Correia EB, Rohde LEP, et al. Posicionamento sobre Diagnóstico e Tratamento da Amiloidose Cardíaca — 2021. *Arq Bras Cardiol*. 2021 Set;117(3):561–598. doi:10.36660/abc.20210718

2. Donnelly JP, Hanna M. Cardiac amyloidosis: an update on diagnosis and treatment. *Cleve Clin J Med*. 2017;84(12 Suppl 3):12-26.

3. Maurer MS, Bokhari S, Damy T, Dorbala S, Drachman BM, Fontana M, et al. Expert consensus recommendations for the suspicion and diagnosis of cardiac ATTR amyloidosis. *Circ Heart Fail*. 2019;12(9):e006075. doi: 10.1161/CIRCHEART FAILURE.119.006075.

4. Ioannou A, Patel RK, Razvi Y, Porcari A, Sinagra G, Venneri L, et al. Impact of earlier diagnosis in cardiac ATTR amyloidosis over the course of 20 years. *Circulation*. 2022;146(22):1657-70. doi:10.1161/CIRCULATIONAHA.122.060852.

5. Kittleson MM, Ruberg FL, Ambardekar AV, Brannagan TH, Cheng RK, Clarke JO, et al. 2023 ACC Expert Consensus Decision Pathway on Comprehensive Multidisciplinary Care for the Patient with Cardiac Amyloidosis: A Report of the American College of Cardiology Solution Set Oversight Committee. J Am Coll Cardiol. 2023;81(11):1076-126. doi: 10.1016/j.jacc.2022.11.022.

6. Maurer MS, Schwartz JH, Gundapaneni B, Elliott PM, Merlini G, Waddington- Cruz M, et al. Tafamidis Treatment for Patients with Transthyretin Amyloid Cardiomyopathy. N Engl J Med. 2018;379(11):1007-16. doi: 10.1056/ NEJMoa1805689.

7. Gillmore JD, Judge DP, Cappelli F, Fontana M, Garcia-Pavia P, Gibbs S, et al. Efficacy and Safety of Acoramidis in Transthyretin Amyloid Cardiomyopathy. N Engl J Med. 2024;390(2):132-42. doi: 10.1056/ NEJMoa2305434.

8. Adams D, Gonzalez-Duarte A, O'Riordan WD, Yang CC, Ueda M, Kristen AV, et al. Patisiran, an RNAi Therapeutic, for Hereditary Transthyretin Amyloidosis. N Engl J Med. 2018;379(1):11-21. doi: 10.1056/ NEJMoa1716153.

9. Benson MD, Waddington-Cruz M, Berk JL, Polydefkis M, Dyck PJ, Wang AK, et al. Inotersen Treatment for Patients with Hereditary Transthyretin Amyloidosis. N Engl J Med. 2018;379(1):22-31. doi: 10.1056/NEJMoa1716793.

10. Judge DP, Kristen AV, Grogan M, Maurer MS, Falk RH, Hanna M, et al. Phase 3 Multicenter Study of Revusiran in Patients with Hereditary Transthyretin- Mediated (hATTR) Amyloidosis with Cardiomyopathy (ENDEAVOUR). Cardiovasc Drugs Ther. 2020;34(3):357-70. doi: 10.1007/s10557-019-06919-4.

11. Maurer MS, Kale P, Fontana M, Berk JL, Grogan M, Gustafsson F, et al. Patisiran Treatment in Patients with Transthyretin Cardiac Amyloidosis. N Engl J Med. 2023;389(17):1553-65. doi: 10.1056/NEJMoa2300757.

12. Fontana M, Berk JL, Gillmore JD, Witteles RM, Grogan M, Drachman B, et al. Vutrisiran in Patients with Transthyretin Amyloidosis with Cardiomyopathy. N Engl J Med. 2025;392(1):33-44. doi: 10.1056/NEJMoa2409134.

13. Solomon SD, Adams D, Kristen A, Grogan M, González-Duarte A, Maurer MS, et al. Effects of patisiran, an RNA interference therapeutic, on cardiac parameters in patients with hereditary transthyretin-mediated amyloidosis: analysis of the APOLLO study. *Circulation*. 2019;139(4):431-43. doi:10.1161/CIRCULATIONAHA.118.035831.

14. Damy T, Garcia-Pavia P, Hanna M, Judge DP, Merlini G, Gundapaneni B, et al. Efficacy and safety of tafamidis doses in the Tafamidis in Transthyretin Cardiomyopathy Clinical Trial (ATTR-ACT) and long-term extension study. *Eur J Heart Fail*. 2021;23(2):277-85. doi:10.1002/ejhf.2064.

15. Miller AB, Januzzi JL, O'Neill BJ, Gundapaneni B, Patterson TA, Sultan MB, et al. Causes of cardiovascular hospitalization and death in patients with transthyretin amyloid cardiomyopathy (from the Tafamidis in Transthyretin Cardiomyopathy Clinical Trial [ATTR-ACT]). *Am J Cardiol*. 2020;132:146-52. doi:10.1016/j.amjcard.2020.07.006.

16. Shah SJ, Fine N, Garcia-Pavia P, Klein AL, Fernandes F, Weissman NJ, Maurer MS, Boman K, Gundapaneni B, Sultan MB, Elliott P. Effect of tafamidis on cardiac function in patients with transthyretin amyloid cardiomyopathy: a post hoc analysis of the ATTR-ACT randomized clinical trial. JAMA Cardiol. 2024;9(1):25-34. doi:10.1001/jamacardio.2023.4310.
