# Supplementary material for: Terapias Modificadoras de Doença para a Cardiomiopatia por Amiloidose por Transtirretina: Uma Revisão Sistemática e Metanálise
Source: Arq Bras Cardiol. 2025 Sep 10;122(8):e20240830. [Article in Portuguese] doi: 10.36660/abc.20240830 (PMC12677762; doi:10.36660/abc.20240830)
Supplement: Supplementary file 2 [file 0066-782X-abc-122-08-e20240830-Suppl02.pdf]

Annex 1

Causes of death listed in any line of the death certificate, grouped by system* and organized according to sex in patients hospitalized with a diagnosis of HF between May 2011 and July 2019.

**Causes mentioned by systems (*)**	Occurrence according to sex, absolute number (%)		Total entries in the DC (%)
**Gender**	Male	Female	
**Circulatory system (I)**	196 (20%)	155 (15.9%)	351 (35.9%)
**Respiratory system (J)**	76 (7.8%)	56 (5.7%)	132 (13.5%)
**Infectious (A and B)**	58 (5.9%)	57 (5.8%)	115 (11.7%)
**Ill-defined (R)**	53 (5.4%)	47 (4.8%)	100 (10.2%)
**Genitourinary system (N)**	54 (5.5%)	35 (3.6%)	89 (9.1%)
**Neoplasms (C and D)**	20 (2.1%)	19 (1.9%)	39 (4.0%)
**Endocrine, nutritional, and metabolic (E)**	22 (2.2%)	14 (1.5%)	36 (3.7%)
**Nervous system and head (F, G, and H)**	13(1.3%	13 (1.3%)	26 (2.6%)
**External causes (V, X, W, and Y)**	16 (1.7%)	14 (1.4%)	30 (3.1%)
**Musculoskeletal and cutaneous system (L and M)**	4 (0.4%)	3 (0.3%)	7 (0.7%)
**Other groups (K, P, O, S, and T)**	27 (2.7%)	25 (2.6%)	52 (5.3%)
**Total**	539 (55.2%)	438 (44.8%)	977 (100%)

Annex 2

Causes of death listed in any line of the death certificate, grouped by system* and organized according to the ejection fraction in patients hospitalized with a diagnosis of HF between May 2011 and July 2019.

**Causes mentioned by system (*)**	Occurrence according to age tertiles, absolute number (%)			Total entries in the DC (%)
**Age (years)**	First Tertile (18 to 55)	Second Tertile (56 to 77)	Third Tertile (78 to 99)	
**Circulatory system (I)**	24 (2.4%)	112 (11.5%)	215 (22%)	351 (35.9%)
**Respiratory system (J)**	5 (0.5%)	35 (3.6%)	92 (9.4%)	132 (13.5%)
**Infectious (A and B)**	7 (0.7%)	38 (3.9%)	70 (7.1%)	115 (11.7%)
**Ill-defined (R)**	3 (0.3%)	27 (2.8%)	70 (7.1%)	100 (10.2%)
**Genitourinary system (N)**	4 (0.4%)	38 (3.9%)	47 (4.8%)	89 (9.1%)
**Neoplasms (C and D)**	1 (0.1%)	23 (2.4%)	15 (1.5%)	39 (4.0%)
**Endocrine, nutritional, and metabolic (E)**	2 (0.2%)	14 (1.5%)	20 (2%)	36 (3.7%)
**Nervous system and head (F, G, and H)**	0 (0%)	5 (0.5%)	21 (2.1%)	26 (2.6%)
**External causes (V, X, W, and Y)**	3 (0.3%)	11 (1.1%)	16 (1.7%)	30 (3.1%)
**Musculoskeletal and cutaneous system (L and M)**	0 (0%)	3 (0.3%)	4 (0.4%)	7 (0.7%)
**Other groups (K, P, O, S, and T)**	6 (0.6%)	22 (2.2%)	24 (2.5%)	52 (5.3%)
**Total**	55 (5.6%)	328 (33.6%)	594 (60.8%)	977 (100%)
