# Supplementary material for: Terapias Modificadoras de Doença para a Cardiomiopatia por Amiloidose por Transtirretina: Uma Revisão Sistemática e Metanálise
Source: Arq Bras Cardiol. 2025 Sep 10;122(8):e20240830. [Article in Portuguese] doi: 10.36660/abc.20240830 (PMC12677762; doi:10.36660/abc.20240830)
Supplement: Translation

# Translation: Disease-Modifying Therapies for Transthyretin Amyloid Cardiomyopathy: A Systematic Review and Meta-Analysis

**DOI:** 10.36660/abc.20240830i

**Authors:** Ligia Carolina Facin, Igor Pacheco Fiuza Romeiro, Kaushiki Sapahia, Beatriz Austregésilo de Athayde de Hollanda Morais, Juliana Queiroz Vasconcelos Muniz, Juliana Domenes Pereira, Cintia Gomes, Celina Borges Migliavaca, André Zimerman, Andreia Biolo

**Affiliations:** 1 Porto Alegre RS Brazil Serviço de Cardiologia, Hospital Moinhos de Vento, Porto Alegre, RS – Brazil; 2 Fortaleza PE Brazil Departamento de Medicina, Universidade Federal do Ceará, Fortaleza, PE – Brazil; 3 Delhi India Department of Medicine, University College of Medical Sciences of New Delhi, Delhi – India; 4 Maceió AL Brazil Departamento de Medicina, Centro Universitário de Maceió (Cesmac), Maceió, AL – Brazil; 5 Heidelberg Germany Department of Internal Medicine, Schmieder Klinik Heidelberg, Heidelberg – Germany; 6 São Paulo SP Brazil Departamento de Clínica Médica, Universidade Estadual de Campinas, São Paulo, SP – Brazil; 7 Colorado USA Department of Internal Medicine, University of Colorado Health Parkview Medical Center, Colorado – USA; 8 Porto Alegre RS Brazil Programa de Pós-Graduação em Cardiologia, Universidade Federal do Rio Grande do Sul, Porto Alegre, RS – Brazil; 9 Porto Alegre RS Brazil MOVE Academic Research Organization, Hospital Moinhos de Vento, Porto Alegre, RS - Brazil; 10 Porto Alegre RS Brazil Serviço de Cardiologia, Hospital de Clínicas de Porto Alegre, Porto Alegre, RS – Brazil

**Keywords:** Transthyretin Amyloid Cardiomyopathy, TTR Stabilizer, TTR Silencer

## Abstract

**Background:**

Transthyretin (TTR) amyloid cardiomyopathy (ATTR-CM) is the most common form of restrictive cardiomyopathy. Emerging pharmacological therapies aim to alter the natural history of disease and delay its advancement. However, data directly comparing the efficacy of different drug classes versus placebo remain limited.

**Objectives:**

This systematic review assessed the efficacy of TTR stabilizers and silencers compared with placebo on all-cause mortality, hospitalizations, functional outcomes, and serum levels of the biomarker NT-proBNP in patients with ATTR-CM.

**Methods:**

A comprehensive search of PubMed, Embase, and Cochrane databases was conducted for randomized controlled trials (RCTs) published through April 2025. Eligible studies compared patisiran, tafamidis, inotersen, revusiran, acoramidis, or vutrisiran to placebo in patients with ATTR-CM. Analyses were stratified by drug class, and statistical significance was set at p<0.05.

**Results:**

Seven RCTs involving 2,526 participants were included; 42.5% received TTR stabilizers and 57.5% received TTR silencers. Compared with placebo, TTR stabilizers significantly reduced all-cause mortality (RR: 0.71; 95% CI 0.59-0.87; p=0.0006) and hospitalizations (RR: 0.81; 95% CI 0.73-0.89; p<0,0001). TTR silencers did not significantly reduce mortality (RR: 0.79; 95% CI 0.37-1.68; p=0.54) or hospitalizations (RR: 1.11; 95% CI 0.83-1.48; p=0.48). Both therapies were associated with improvements in 6-minute walk distance, quality of life, and reductions in serum NT-proBNP levels.

**Conclusion:**

TTR stabilizers significantly reduced all-cause mortality and hospitalizations in patients with ATTR-CM compared with placebo. These benefits were not observed with TTR silencers, potentially due to shorter follow-up durations in the studies evaluated. Both therapies improved functional status and serum levels of NT-proBNP.

## Highlights

- Disease-modifying therapies for ATTR-CM are available and can slow disease progression and improve prognosis;
- In this meta-analysis of RCTs, TTR stabilizers significantly reduced all-cause mortality and hospitalizations, confirming their efficacy in ATTR-CM;
- TTR silencers did not show significant effects on mortality or hospitalizations compared with placebo; these findings may be influenced by shorter follow-up durations in the available studies;
- Ongoing trials of TTR silencers and emerging drug classes are expected to provide additional evidence and complement the findings of this meta-analysis.

**Figure:** TTR stabilizers prevent the dissociation of the transthyretin protein, while TTR silencers inhibit its production, primarily within the cytoplasm of liver cells. Data indicate that TTR stabilizers significantly reduce all-cause mortality compared with placebo in patients with ATTR-CM. ATTR-CM: transthyretin amyloid cardiomyopathy; Chi^2^: chi-square; CI: confidence interval; df: degrees of freedom; I^2^: Higgins’ I^2^statistics; M-H: Mantel-Haenszel; p: p-value; Tau: Kendall's tau; TTR: transthyretin.

## Introduction

Cardiac amyloidosis (CA) has an estimated incidence of 18 to 55 cases per 100,000 person-years, with its prevalence and associated mortality rising steadily over recent decades.^1^In the United Kingdom, the number of patients diagnosed with amyloidosis has increased 6.7-fold since the 1990s, likely reflecting improved disease awareness and advances in diagnostic techniques. In the United States, mortality rates have also risen significantly, from 1.77 per 1,000,000 in 1979 to 3.96 per 1,000,000 in 2015.^2^CA is a progressive condition that can be fatal if not diagnosed and treated promptly.^3 - 5^

Conventional therapies target clinical manifestations such as arrhythmias or heart failure but are not disease-modifying.^6 , 7^The development of disease-modifying therapies has transformed the management of transthyretin (TTR) amyloid cardiomyopathy (ATTR-CM). TTR is a short-lived soluble plasma protein, primarily synthesized by the liver, with a tetrameric structure composed of four identical subunits responsible for transporting thyroxine and vitamin A. The most widely studied and clinically available therapies targeting amyloid cardiomyopathy are TTR stabilizers and TTR silencers ( Central Illustration ).^8 - 10^TTR stabilizers, including tafamidis and acoramidis, bind to the dimer-dimer interface of the TTR tetramer, preventing its dissociation into monomers and, consequently, the formation of amyloid fibrils that accumulate in the myocardium.^11^TTR silencers, on the other hand, act primarily within the cytoplasm of hepatocytes by inhibiting TTR protein synthesis, significantly reducing circulating TTR concentrations.^12^This class includes antisense oligonucleotides (e.g., inotersen) and small interfering RNAs (e.g., revusiran, patisiran, and vutrisiran).

To date, no study has comprehensively synthesized the effectiveness of ATTR-targeted therapies, particularly when stratified by mechanism of action.^13 , 14^This review aimed to evaluate the efficacy and safety of disease-modifying therapies for ATTR-CM, including tafamidis, revusiran, patisiran, inotersen, acoramidis, and vutrisiran, compared with placebo. The analysis focused on patient-centered outcomes, including all-cause mortality and hospitalizations, functional capacity, quality of life, and serum NT-proBNP levels.

## Methods

### Protocol

This review was prospectively registered in the International Prospective Register of Systematic Reviews (PROSPERO) under protocol number CRD42024517136.

### Study design

This review was conducted in accordance with the Cochrane Handbook for Systematic Reviews of Interventions and reported following the Preferred Reporting Items for Systematic Reviews and Meta-Analyses (PRISMA).^15 , 16^

### Search strategy, eligibility criteria, and data extraction

A systematic search of PubMed, Embase, and Cochrane databases was performed on April 13, 2025. The complete search strategy is provided in Table S1 . Following the removal of duplicates, study selection was conducted in two phases. First, titles and abstracts were screened using Zotero. Subsequently, the full texts of potentially eligible studies were assessed for inclusion. Two independent review authors (L.F. and C.G.) performed the selection process, with discrepancies resolved by a third review author (A.B.).

Inclusion criteria (1) peer-reviewed randomized controlled trials (RCTs); (2) evaluation of disease-modifying therapies for ATTR-CM in patients with confirmed cardiac involvement; and (3) reporting at least one outcome of interest. Studies were excluded if they (1) included duplicate populations; (2) lacked a control group; or (3) did not report relevant outcomes. No restrictions were applied regarding publication date, language, or follow-up duration.

Data extracted included study design (follow-up duration, intervention, and number of patients randomized) and baseline patient characteristics (sex, age, New York Heart Association [NYHA] functional class, N-terminal pro-B-type natriuretic peptide [NT-proBNP], and left ventricular [LV] ejection fraction [LVEF]).

Two independent review authors (L.F. and I.P.) performed data extraction using a pre-designed Excel sheet developed for this review.

### Outcomes of interest

The outcomes of interest included all-cause mortality, cardiovascular mortality, all-cause hospitalizations, cardiovascular hospitalizations, heart failure hospitalizations, functional capacity assessed by the 6-minute walk test (6-MWT), quality of life assessed by the Kansas City Cardiomyopathy Questionnaire Overall Score (KCCQ-OS), serum NT-proBNP and TTR levels, LV global longitudinal strain (GLS), LV mass, LV wall thickness, and LVEF. The availability of each outcome across the included studies is detailed in Table S2 .

### Data analysis and statistics

Random-effects meta-analyses were performed using the Mantel-Haenszel or Generic Inverse Variance method for binary outcomes and the inverse variance method for continuous outcomes, with Tau estimated using the DerSimonian and Laird approach.^16^When necessary, standard deviations for change were imputed using a correlation coefficient of 0.5.^16^Heterogeneity was assessed using the Higgins’ I^2^statistics and further explored through subgroup analyses.

All statistical analyses were conducted using RevMan version 5.4 (Nordic Cochrane Centre, The Cochrane Collaboration, Copenhagen, Denmark).^17^A two-sided p-value <0.05 was considered statistically significant.

Additionally, to account for variations in follow-up duration, annualized changes in 6-MWT and KCCQ-OS were calculated by dividing the absolute change by the follow-up time reported in each study.

### Quality assessment

The risk of bias in the included studies was assessed using the Risk of Bias 2 (RoB 2) tool for RCTs.^18^The certainty of evidence for each outcome was evaluated using the Grading of Recommendations, Assessment, Development and Evaluation (GRADE) approach.^19^

## Results

### Study selection and baseline characteristics

The study selection process is illustrated in Figure 1 . A total of 2.504 references were identified through database searches. After removing duplicates and screening titles and abstracts, 44 articles were selected for full-text review. Ultimately, seven RCTs were included, reported across 11 publications, including four post hoc analyses. These trials enrolled a total of 2,526 patients, with 1,073 (42.5%) included in studies evaluating TTR stabilizers and 1,453 (57.5%) in studies evaluating TTR silencers.

**Figure 1:** PRISMA flow diagram for study screening and selection.

Baseline patient characteristics are summarized in Table 1 . The seven included RCTs evaluated patisiran, inotersen, tafamidis, revusiran, acoramidis, and vutrisiran. Most participants were male (89%). Across studies, the mean or median LVEF was consistently ≥50%, and median NT-proBNP levels were uniformly elevated. Additionally, most patients (86%) were classified as NYHA functional class I or II. Follow-up durations ranged from approximately 6 to 36 months.

**Table 1:** Baseline characteristics of the included studies

Study ID	Intervention	Main population	TTR class	Patients (Int/Pla)	Age a (Int/Pla)	Female(%)(Int/Pla)	NYHA < III(%)(Int/Pla)	NT-proBNP b (pg/mL)(Int/Pla)	LVEF a (%)(Int/Pla)	Follow-up period
Adams 2018 (APOLLO)^12^	Patisiran	ATTR-PN	Silencer	90/36	60/62	24/18	100/100	756.4/845.7	60/62	18 months
Benson 2018 (NEURO-TTR)^28^	Inotersen	ATTR-PN	Silencer	75/33	NA d	NA d	100/100	NA d	65/64	15 months
Maurer 2018 (ATTR-ACT)^22^	Tafamidis	ATTR-CM	Stabilizer	264/177	74/74	9/11	70/65	2.995.9/3.161.0	48/49	30 months
Judge 2020 (ENDEAVOUR)^20^	Revusiran	ATTR-CM	Silencer	140/66	69/68	25/20	69/70	2.371.0/2.719.0	53/51	6.7 months c
Maurer 2023 (APOLLO-B)^29^	Patisiran	ATTR-CM	Silencer	181/178	76/76	11/10	92/93	2.008.0/1.813.0	58/60	12 months
Gilmore 2024 (ATTRibute-CM)^21^	Acoramidis	ATTR-CM	Stabilizer	421/211	77/77	9/12	89/90	2.326.0/2.306.0	NA e	30 months
Fontana 2024 (HELIOS-B)^23^	Vutrisiran	ATTR-CM	Silencer	326/328	77/76	8/7	92/90	2.021.0/1.801.0	NA e	36 months

a Median or mean;

b Median;

c Median;

d No data available for patients with cardiomyopathy;

e Data not available.

ATTR-PN: transthyretin amyloidosis with polyneuropathy; ATTR-CM: transthyretin amyloid cardiomyopathy; Int: intervention group; Pla: placebo group; NYHA: New York Heart Association; NT-proBNP: N-terminal pro-B-type natriuretic peptide; LVEF: left ventricular ejection fraction; NA: not available. All studies adopted a statistical significance level of 5% (p < 0.05).

### Quality assessment

Overall, most outcomes were classified as having a low risk of bias. However, several outcomes from the ENDEAVOUR trial^20^were rated as high risk of bias or as having some concerns, primarily due to issues related to outcome measurement and missing data. The detailed risk of bias assessment is shown in Figure S1 .

The certainty of evidence for each outcome was rated as very low, low, moderate, or high, as presented in Table S3 . The most frequent limitations identified were inconsistency and imprecision.

### Mortality and hospitalizations

As shown in Figure 2A , when all studies were pooled, there was a significant reduction in all-cause mortality (RR: 0.72; 95% CI, 0.57-0.91; p=0.005). In the subgroup analysis, TTR stabilizers significantly reduced all-cause mortality compared with placebo (RR: 0.71; 95% CI 0.59-0.87; p=0.0006), whereas no significant difference was observed in the TTR silencers subgroup (RR: 0.79; 95% CI 0.37-1.68; p=0.54). Regarding cardiovascular mortality, available data were limited to studies evaluating TTR silencers, which did not demonstrate a significant reduction in risk compared with placebo (RR: 1.87; 95% CI 0.64-5.44; p=0.25). Despite these findings for all-cause mortality, no significant differences were observed between drug classes and placebo when analyzing p for interaction.

**Figure 2:** A) All-cause mortality with disease-modifying therapies versus placebo in patients with ATTR-CM. B) All-cause hospitalization with disease-modifying therapies versus placebo in patients with ATTR-CM. Chi^2^: chi-square; CI: confidence interval; df: degrees of freedom; I^2^: Higgins’ I^2^statistics; IV: inverse variance; log: logarithm; M-H: Mantel-Haenszel; p: p-value; SE: standard error; Tau: Kendall's tau; TTR: transthyretin.

As shown in Figure 2B , when all therapies were combined, there was no significant effect on all-cause hospitalizations (RR: 0.90; 95% CI 0.77-1.06; p=0.21). However, in the subgroup analysis, TTR stabilizers significantly reduced hospitalization rates compared with placebo (RR: 0.81; 95% CI 0.73-0.89; p<0.0001), while TTR silencers showed no significant effect (RR: 1.11; 95% CI 0.83-1.48; p=0.48). For cardiovascular hospitalizations, meta-analysis by therapeutic class was not feasible; the analysis was limited to all therapies combined and did not demonstrate a statistically significant effect (RR: 0.90; 95% CI: 0.75–1.09; p = 0.28). Regarding heart failure hospitalizations, meta-analysis was feasible only for the comparison of all therapies combined (RR: 1.03; 95% CI: 0.69–1.54; p = 0.88) and for the TTR silencers subgroup (RR: 1.40; 95% CI: 0.84–2.34; p = 0.19), with neither showing significant differences.

### Functional capacity and quality of life

As shown in Figure 3A , treatment with disease-modifying therapies significantly improved the 6-minute walk distance (6-MWD) compared with placebo (mean difference [MD] 31.81 meters; 95% CI, 10.99-52.62; p=0.003). Similarly, Figure 3B shows a significant improvement in KCCQ-OS scores (MD 8.05 points; 95% CI, 3.99-12.10; p<0.0001).

**Figure 3:** (A) 6-minute walk distance (6-MWD) with disease-modifying therapies versus placebo in patients with ATTR-CM. B) Kansas City Cardiomyopathy Questionnaire Overall Score (KCCQ-OS) with disease-modifying therapies versus placebo in patients with ATTR-CM. 6-MWD: 6-minute walk distance; Chi^2^: chi-square; CI: confidence interval; df: degrees of freedom;I^2^: Higgins’ I^2^statistics; IV: inverse variance; p: p-value; Tau: Kendall's tau; TTR: transthyretin; KCCQ-OS: Kansas City Cardiomyopathy Questionnaire Overall Score.

In the subgroup analysis, the improvement in 6-MWD was significant in both groups: TTR stabilizers (MD 57.16 meters; 95% CI, 21.92-92.40; p=0.001) and TTR silencers (MD 18.02 meters; 95% CI, 4.22-31.82; p=0.01). Regarding KCCQ-OS, both subgroups also showed significant improvements: TTR stabilizers (MD 11.55 points; 95% CI, 7.89-15.22; p<0.00001) and TTR silencers (MD 4.82 points; 95% CI, 2.39-7.25; p<0.0001).

### Laboratory endpoints

As shown in Figure 4A , treatment with disease-modifying therapies resulted in a significant reduction in NT-proBNP levels compared with placebo (mean difference [MD] in geometric change from baseline: −0.86 pg/mL; 95% CI −1.30 to −0.41; p=0.0002). Similarly, Figure S2 demonstrates a significant reduction in serum TTR protein levels compared with placebo (MD: −70.25%; 95% CI −97.04 to −43.46; p<0.00001).

**Figure 4:** Serum NT-proBNP levels with disease-modifying therapies versus placebo in patients with ATTR-CM. Chi^2^: chi-square; CI: confidence interval; df: degrees of freedom; I^2^: Higgins’ I^2^statistics; IV: inverse variance; NT-proBNP: N-terminal pro-B-type natriuretic peptide; p: p-value; Tau: Kendall's tau; TTR: transthyretin.

In the subgroup analysis by therapy class, both TTR stabilizers (MD in geometric change from baseline: −1.42 pg/mL; 95% CI −1.67 to −1.16; p<0.00001) and TTR silencers (MD in geometric change from baseline: - 0.42 pg/mL; 95% CI - 0.70 to - 0.15; p=0.002) significantly reduced NT-proBNP levels. Regarding serum TTR levels, meta-analysis was only feasible for the TTR silencer subgroup, which showed a significant reduction (MD: –81.62%; 95% CI: –86.44 to –76.80; p < 0.00001).

### Echocardiographic endpoints

Figures S3A to S3D present the results of the echocardiographic outcome analyses. Although all outcomes were evaluated in the overall analysis, only studies investigating TTR silencers provided sufficient data for subgroup meta-analysis.

The use of disease-modifying therapies was associated with a significant improvement in LV GLS ( Figure S3A ) compared with placebo (MD: - 0.83%; 95% CI: −1.27 to −0.40; p = 0.0002). Subgroup analysis demonstrated statistical significance among TTR silencers (MD: - 0.85%; 95% CI: −1.41 to - 0.30; p = 0.003).

Regarding LV mass ( Figure S3B ), a significant reduction was observed compared with placebo (MD: −9.74 g; 95% CI: −17.07 to -2.40; p = 0.009). This outcome was reported exclusively in studies evaluating TTR silencers.

No statistically significant differences in LV wall thickness ( Figure S3C ) were identified in the overall analysis (MD: - 0.30 mm; 95% CI: - 0.66 to 0.06; p = 0.10) or within the TTR silencer subgroup (MD: - 0.28 mm; 95% CI: −0.67 to 0.11; p = 0.16).

Similarly, no significant differences in LVEF ( Figure S3D ) were observed between all intervention groups and placebo (MD: −0.16%; 95% CI: –1.97 to 1.64; p = 0.86) or within the TTR silencer subgroup (MD: –0.99%; 95% CI: –3.05 to 1.06; p = 0.34).

### Sensitivity analysis

A sensitivity analysis excluding the ENDEAVOUR trial^20^— due to its premature termination — was conducted. This analysis revealed a significant reduction in all-cause mortality (RR: 0.70; 95% CI 0.60-0.82; p<0.0001) ( Figure S4A ). In the subgroup analysis, both TTR stabilizers (RR: 0.71; 95% CI 0.59-0.87; p=0.0006) and TTR silencers (RR: 0.67; 95% CI 0.51-0.89; p=0.005) demonstrated a significant reduction in mortality compared with placebo.

Despite this finding, no significant difference between the drug class and placebo was observed in the sensitivity analysis assessing the p-value for interaction.

In the analysis of left ventricular wall thickness, exclusion of the ENDEAVOUR^20^trial resulted in a significant reduction for the combined therapeutic classes (MD –0.44 mm; 95% CI –0.81 to –0.06; p=0.02) and for the TTR silencers subgroup (MD –0.44 mm; 95% CI –0.84 to –0.03; p=0.03).

A sensitivity analysis was also conducted, excluding studies in which the primary objective was the treatment of amyloid neuropathies. The APOLLO^12^trial was excluded from the analyses of the main outcomes—all-cause mortality ( Figure S5A ) and serum NT-proBNP ( Figure S5B )—to confirm that its exclusion did not affect the statistical significance. For all-cause mortality, the overall analysis remained significant (RR 0.73; 95% CI 0.57–0.94; p=0.01), whereas no significant effect was observed in the TTR silencers subgroup (RR 0.92; 95% CI 0.33–2.57; p=0.88). For NT-proBNP, statistical significance was preserved in the overall analysis (MD in geometric change from baseline –0.89 pg/mL; 95% CI –1.39 to –0.40; p=0.0004) as well as in the TTR silencers subgroup (MD in geometric change from baseline –0.40 pg/mL; 95% CI –0.70 to –0.10; p=0.010). The NEURO-TTR^28^trial was not included in this assessment, as it reported only echocardiographic data.

## Discussion

This review included seven RCTs comparing various disease-modifying therapies with placebo in patients with ATTR-CM. The main findings were as follows: (1) TTR stabilizers significantly reduced all-cause mortality and hospitalizations compared with placebo; (2) TTR silencers did not demonstrate a significant reduction in these outcomes; and (3) both therapy classes were effective in improving functional capacity (6-MWD) and quality of life (KCCQ-OS),as well as in reducing serum NT-proBNP levels compared with placebo.

We observed that TTR stabilizers significantly reduced mortality compared with placebo. Although the ATTRibute trial^21^did not show a statistically significant reduction in mortality, the authors suggested this may be attributed to increased disease awareness, leading to earlier diagnosis and treatment, and consequently, a lower all-cause mortality risk in the study population. When combining the results of the ATTR-ACT^22^and ATTRibute^21^trials in our review, the increased statistical power revealed a consistent reduction in mortality risk across both studies. Furthermore, the beneficial effects of TTR stabilizers on mortality have also been demonstrated in real-world settings, supporting the external validity of these findings.^11^

The lack of a significant effect of TTR silencers on mortality may be partly attributed to the shorter follow-up periods in most of the included studies, leading to fewer observed events compared with trials evaluating TTR stabilizers. In studies of TTR stabilizers, differences in mortality between the intervention and placebo groups typically emerged between 15 and 18 months, with both RCTs having follow-up durations of 30 months. In contrast, the average follow-up in TTR silencer trials was approximately 17 months. Notably, the HELIOS-B trial,^23^which had a longer follow-up of 36 months, did demonstrate a significant reduction in mortality. Furthermore, when performing a sensitivity analysis excluding the prematurely terminated ENDEAVOUR trial,^20^TTR silencers also showed a significant reduction in all-cause mortality compared with placebo.

Hospitalizations represent a critical burden in the management of heart failure. In this analysis, we pooled data on all-cause, cardiovascular, and heart failure-related hospitalizations. Our findings demonstrated a significant reduction in all-cause hospitalizations among patients treated with TTR stabilizers compared with placebo. However, variations in the classification of hospitalization events across the included RCTs limited the ability to standardize these data for a more definitive conclusion.

To account for the differing follow-up durations — longer in trials evaluating TTR stabilizers — the placebo-adjusted annual change in 6-MWD was calculated ( Table S4 ). When considering such results, both therapy classes demonstrated comparable improvements. However, the adjusted improvements in 6-MWD remained below the threshold of 35 meters per year, which has been suggested to have prognostic significance in CA.^24^Similarly, for KCCQ-OS, the placebo-adjusted annual change were comparable between therapy classes. Nevertheless, TTR stabilizers achieved a greater improvement in the ATTR-ACT trial^22^surpassing the 5-point threshold that has been associated with clinically meaningful and prognostic impact.^25^

Regarding biomarkers, our overall analysis demonstrated that disease-modifying therapies were associated with significant reductions in serum NT-proBNP and TTR protein levels, both routinely used to assess disease severity. TTR stabilizers resulted in a greater reduction in NT-proBNP, while TTR silencers led to a more pronounced decrease in serum TTR levels, consistent with their mechanism of transcriptional inhibition of hepatic TTR synthesis. Although TTR stabilizers are generally expected to preserve or increase circulating TTR levels by preventing tetramer dissociation, the only available data in our analysis came from the ATTRibute-CM trial,^21^which reported a reduction. This result therefore warrants cautious interpretation.

The applicability of echocardiographic parameters in assessing the prognosis of CA is limited, as most data derive from small-scale studies. However, particularly in asymptomatic patients, echocardiography has proven useful in detecting early disease progression.^27^Our review demonstrated significant improvement in LV GLS both in the overall analysis and in the TTR silencers subgroup. Regarding LV mass, a significant reduction was observed exclusively in TTR silencers subgroup. No significant effects were found on LV wall thickness or LVEF, either in the overall analysis or in TTR silencers subgroup. However, in the sensitivity analysis excluding the ENDEAVOUR^20^trial, a statistically significant reduction in left ventricular wall thickness was observed for both the combined therapeutic classes and for TTR silencers versus placebo. It is important to note that the sample size for echocardiographic outcomes was limited in most analyses.

Previous meta-analyses^13 , 14^have primarily focused on the effectiveness of tafamidis, incorporating data from both RCTs and observational studies. In contrast, our analysis included seven RCTs, five of which were specifically designed for ATTR-CM. Similarly, a meta-analysis by Wang et al.^13^assessed the effect of tafamidis in ATTR-CM and reported a significant association between TTR stabilizer therapy and reduced mortality, consistent with the findings of our study.

This review has several limitations. First, although the APOLLO^12^and NEURO-TTR^28^trials were primarily designed to assess neurological outcomes, only cardiomyopathy-related data were included in our analysis. A sensitivity analysis excluding the APOLLO trial confirmed that its exclusion did not alter the results. Second, the shorter follow-up durations in trials of TTR silencers may have limited the number of clinical events; to address this, we evaluated the placebo-adjusted annual change ( Table S4 ). Third, the early termination of the ENDEAVOUR trial^20^may have compromised the reliability of its data; thus, a sensitivity analysis excluding this trial is presented in the supplementary analyses. Fourth, we did not perform direct comparisons between TTR stabilizers and silencers, as all comparisons were made against placebo. Finally, echocardiographic data remain limited across trials; however, ongoing studies are expected to provide more robust evidence.

This study also has several strengths, including its rigorous methodology, strict adherence to Cochrane and PRISMA guidelines, and the comprehensive inclusion of all available RCTs evaluating the efficacy of currently approved therapies for ATTR-CM.

## Conclusion

In this systematic review and meta-analysis of disease-modifying therapies for CA, TTR stabilizers significantly reduced all-cause mortality and hospitalizations in patients with ATTR-CM compared with placebo. In contrast, TTR silencers did not demonstrate a significant impact on these outcomes relative to placebo. Additional studies are required to establish the long-term efficacy of this therapeutic class.

Data Availability

The underlying content of the research text is contained within the manuscript.

## *Supplemental Materials

For additional information, please click here
